# Psychometric evaluation of the Palliative Nursing Care Quality Scale (PNCQS) among ICU and oncology nurses in Iran

**DOI:** 10.1371/journal.pone.0361384

**Published:** 2026-11-04

**Authors:** Mehraban Shahmari, Sevda Gardashkhani, Elaheh Habibpour, Ehsan Kamalifar, Nahid Dehghan Nayeri, Mehran Zare

**Affiliations:** 1 Department of Medical-Surgical Nursing, School of Nursing and Midwifery, Ardabil University of Medical Sciences, Ardabil, Iran; 2 School of Nursing and Midwifery, Tabriz University of Medical Sciences, Tabriz, Iran; 3 Department of Medical-Surgical Nursing, Faculty of Nursing and Midwifery, Tabriz University of Medical Sciences, Tabriz, Iran; 4 Department of Medical-Surgical Nursing, School of Nursing and Midwifery, Ardabil University of Medical Sciences, Ardabil, Iran; 5 School of Nursing & Midwifery, Tehran University of Medical Sciences, Tehran, Iran; 6 Nursing and Midwifery Care Research Center, School of Nursing and Midwifery, Tehran University of Medical Sciences, Tehran, Iran; 7 Al-Subtain University of Medical Sciences, International Branch of Tehran University of Medical Sciences in Iraq, Karbala, Iraq; 8 School of Nursing and Midwifery, Tabriz University of Medical Sciences, Tabriz, Iran; Khoy University of Medical Sciences, IRAN, ISLAMIC REPUBLIC OF

## Abstract

**Background:**

Reliable and culturally adapted instruments are essential for assessing the quality of palliative nursing care. The Palliative Nursing Care Quality Scale (PNCQS) had not been psychometrically evaluated in Iran. This study aimed to translate the PNCQS into Persian and examine its validity and reliability among Iranian nurses.

**Methods:**

In this methodological study, the PNCQS was translated into Persian according to the World Health Organization (WHO) guidelines. A total of 620 nurses from oncology and intensive care units in teaching hospitals in Tehran, Tabriz, and Ardabil were recruited using a multi stage cluster sampling strategy with convenience sampling at the final stage. Face, content, and construct validity were evaluated. Construct validity was examined by first testing the original one-factor model using confirmatory factor analysis (CFA), followed by exploratory factor analysis (EFA) and cross-validation of the derived factor structure using CFA on an independent sample. CFA was conducted in AMOS using maximum likelihood estimation with bootstrapping (1,000 resamples). Reliability was assessed by internal consistency (Cronbach’s α) and two-week test–retest reliability using the intraclass correlation coefficient (ICC).

**Results:**

The scale showed excellent content validity (S-CVI = 0.99). The initial CFA demonstrated that the original 20-item unidimensional model did not adequately fit the Iranian data. Subsequent EFA identified a four-factor structure—Family-Centered Care, Empathic and Spiritual Care, Hope and Life Review, and Physical Symptom Management—explaining 51.41% of the total variance. This structure was confirmed by CFA in an independent sample (n = 400), demonstrating good model fit (χ²/df = 2.84, CFI = 0.95, TLI = 0.95, RMSEA = 0.06). Composite reliability exceeded 0.70 for all factors. Although the AVE for Family-Centered Care was slightly below the recommended threshold (0.42), its composite reliability was acceptable (0.81), and discriminant validity was established for all constructs. Cronbach’s α was 0.92 for the total scale (subscales: 0.76–0.83), and the two-week test–retest ICC was 0.76.

**Conclusion:**

The Persian PNCQS demonstrated satisfactory psychometric properties and a culturally appropriate four-factor structure for assessing the quality of palliative nursing care among Iranian nurses. It provides a reliable multidimensional instrument for clinical practice, research, and quality improvement initiatives in palliative care.

## 1. Introduction

Palliative care is a comprehensive health approach focused on alleviating symptoms and improving the quality of life for patients with serious illnesses and their families. This care addresses physical, psychological, social, and spiritual needs, effectively reducing symptom burden and enhancing patients’ perceptions of quality of life. With the global increase in elderly populations and chronic diseases, the demand for palliative care is rapidly growing [1–3]. With over 20.4 million people worldwide requiring palliative care—of whom 19 million are adults and 34% are cancer patients—this highlights the critical need for culturally and ethnically sensitive palliative care services [4].

As an essential component of healthcare systems, palliative care is provided based on patient needs rather than prognosis and is appropriate at any age and stage of serious illness [5–7]. Delivered by multidisciplinary teams including physicians, nurses, and other trained professionals [8,9], its primary goal is to improve the quality of life for both patients and their families through pain management, symptom control, and psychosocial and spiritual support [5,6,8,10]. Accordingly, these services not only support patients but also assist families in coping with the challenges of serious illness [1]. It should be pointed out that nurses play a pivotal role in delivering this care due to their direct patient contact and ability to effectively manage symptoms and enhance quality of life [6].

Palliative care is indispensable within healthcare systems, significantly improving the quality of life for patients and their families. Conversely, lack of access to such care results in unmanaged distressing symptoms, diminished quality of life, increased psychological burden on families, and elevated healthcare costs due to prolonged hospital stays and intensive treatments [11].

Given the importance of palliative care, evaluating its quality is crucial to enhance service delivery and ensure that patients and their families receive optimal care. Valid and reliable measurement tools are essential for this purpose [12,13]. Among existing instruments, the Palliative Nursing Care Quality Scale (PNCQS), developed by Zulueta Egea et al. (2020), offers a valuable framework for systematically assessing nursing care quality and guiding nursing performance indicators [14].

Despite the recognized importance of palliative care and the availability of assessment tools, there is a notable lack of validated instruments tailored to the Iranian context. Existing scales, such as the Persian version of the Palliative Outcome Scale (POS) for adult cancer patients, have demonstrated acceptable psychometric properties but are limited to specific populations (primarily cancer patients) and do not comprehensively evaluate nurse-driven care quality across various serious illnesses [15]. Moreover, most available tools focus on patient or family outcomes rather than the quality of nursing care delivery itself. This gap underscores the need for nurse-centered, culturally adapted instruments capable of assessing the multifaceted nature of palliative nursing care in Iran.

Although the original PNCQS was designed as a unidimensional scale [14], cross-cultural adaptation of health measurement tools extends beyond translation and may fundamentally affect construct validity [16,17]. Perceptions of care quality, particularly in palliative contexts, are strongly influenced by socio-cultural norms [18,19]. Unlike Western individualistic societies where palliative care quality is often viewed as a single clinical outcome, Iranian culture is deeply rooted in collectivism, family involvement, and spiritual-religious values [20–23]. These cultural attributes may prompt nurses to perceive care quality through distinct yet interconnected dimensions—such as separating family psychological support from physical symptom management, or viewing spiritual care as independent from general psychosocial support. Accordingly, an instrument that is unidimensional in Spain may reveal a multidimensional structure in Iran, reflecting the complex nature of care within a family-oriented and spiritually rich healthcare system [24]. Hence, we hypothesized that the Persian version of the PNCQS would exhibit a multidimensional factor structure and aimed to identify the most appropriate model through exploratory and confirmatory factor analyses for the Iranian nursing context.

The increasing demand for palliative care in Iran [20], coupled with the absence of culturally adapted, psychometrically validated instruments like the PNCQS, highlights a critical gap. Challenges in Iran’s palliative care delivery, including fragmented services and lack of structured frameworks, further emphasize the need for reliable evaluation tools [20,25]. Supporting nurses through validated instruments is vital to improving care quality and addressing the psychological and spiritual needs that are particularly significant within Iranian culture [23].

This study aimed to address this gap by conducting a psychometric evaluation of the Persian version of the Palliative Nursing Care Quality Scale (PNCQS) among nurses in Iran. The findings contribute to the development and refinement of assessment tools, ensuring their appropriate use in clinical practice and research. Moreover, the results inform future studies and support the design of educational and policy initiatives aimed at strengthening palliative nursing care in Iran. Therefore, this research was conducted to validate the Persian PNCQS in order to enhance the quality assessment of palliative nursing care across diverse patient populations and healthcare settings in Iran.

## 2. Materials and methods

### 2.1 Design and setting

This methodological study was conducted in 2025 to evaluate the psychometric properties of the Persian version of the Palliative Nursing Care Quality Scale (PNCQS) among nurses working in oncology and intensive care units in teaching hospitals affiliated with the University of Medical Sciences in Ardabil, Tabriz, and Tehran, Iran. The study assessed the scale’s validity, including face, content, and construct validity (using exploratory and confirmatory factor analysis, as well as convergent validity), and its reliability (internal consistency and test-retest reliability). The translation process followed the World Health Organization’s (WHO) guidelines, and the assessment of measurement properties adhered to the COSMIN checklist to ensure methodological rigor [16].

#### 2.1.1 Participants and sample size.

The study population consisted of nurses working in intensive care units (ICUs) and oncology units in teaching hospitals affiliated with universities of medical sciences in Tehran, Tabriz, and Ardabil, Iran. To achieve a representative sample of the target population, a multi-stage cluster sampling method was employed.

**Sampling procedure:**

**Stage 1 – Primary clusters (cities):** The three cities (Tehran, Tabriz, Ardabil) were considered as primary clusters. All were included to ensure geographical diversity.
**Stage 2 – Secondary clusters (hospitals):** From each city, teaching hospitals with active ICUs and oncology units were listed. Using random sampling, approximately 20 hospitals were selected proportionally across the three cities.
**Stage 3 – Tertiary clusters (wards and nurses):** Within each selected hospital, ICUs and oncology wards were identified. Nurses who met the inclusion criteria were then recruited using convenience sampling within these wards. This approach was necessitated by practical constraints, including rotating shift schedules, high workload of nurses, and limited researcher access to the wards during peak clinical hours.

Although multi-stage cluster sampling was employed at the higher levels to enhance representativeness, the use of convenience sampling at the final stage may have introduced selection bias, potentially leading to the over-representation of more available or motivated nurses. Consequently, the generalizability of the findings to the broader population of nurses working in oncology and ICU settings in Iran may be somewhat limited. This issue is further addressed in the Limitations subsection of the Discussion.

### Sample size determination

Sample size was calculated based on the rule of participants per item for factor analysis [19].

- For an initial confirmatory factor analysis (CFA) of the original unidimensional model, a minimum of 10 participants per item is recommended. Given the original scale contains 20 items, the required sample was 200. Accounting for a 10% attrition rate, the initial target was 220 participants. This sample (n = 220) was first used to test the fit of the original one-factor model proposed by Zulueta Egea et al. (2020).
- Since the original unidimensional model demonstrated poor fit (as detailed in the Results section), we subsequently performed exploratory factor analysis (EFA) on the same sample to identify the underlying factor structure in the Iranian nursing context. This sequential approach (initial CFA followed by EFA on the same sample) is a common and efficient practice in psychometric studies when testing whether an established model transfers to a new cultural setting before exploring alternative structures [26].
- Although using the same sample for both the initial CFA and EFA is not ideal and may capitalize on sample-specific characteristics, it was practically justified in this study due to resource constraints and the clear theoretical rationale for first evaluating the original model. To mitigate this limitation and ensure robust validation of the newly derived four-factor structure, a completely independent sample (n = 400) was used for the final confirmatory factor analysis (CFA). This two-step validation strategy strengthens the overall findings. Test-retest reliability was assessed on a separate convenience subsample of 30 nurses.

In total, **620 nurses** participated in the study. These calculations are consistent with Chow et al. (2017) [27], who considered a 10–20% attrition rate acceptable in clinical studies.

**Inclusion criteria** were: (1) holding at least a bachelor’s degree in nursing; (2) having more than six months of work experience in special wards (ICU) or oncology; (3) providing informed consent. Participants who did not fully complete the questionnaire were excluded.

#### 2.1.2 Data collection.

Data collection took place from August 2025 to April 2026. Eligible nurses, selected via the multi‑stage cluster sampling procedure described earlier, were invited to participate. After providing written informed consent, participants completed a demographic information form and the Persian version of the Palliative Nursing Care Quality Scale (PNCQS) is provided as S1 Questionnaire. The questionnaires were self‑administered. To prevent missing data, each questionnaire was checked for completeness immediately after completion. No identifying information was recorded on the forms; instead, a unique code was assigned to each participant to ensure anonymity.

The instruments used in this study included:

*Demographic Information Form:*

This form included age, gender, marital status, educational level, work experience, city of service, ward of service, and employment status (permanent and Temporary).

*Palliative Nursing Care Quality Scale (PNCQS):*

The main instrument used in this study was the PNCQS, developed by Zulueta Egea et al. (2020) in Spain. In the original study, the scale was unidimensional with 20 items. Items were rated on a five‑point Likert scale ranging from 1 (Almost Never) to 5 (Almost Always), with total scores ranging from 20 to 100. Higher scores indicated better quality of palliative nursing care. The reliability of the original tool was reported with a Cronbach’s alpha of 0.94 [14].

#### 2.1.3 Ethical considerations.

Ethical approval for this study was obtained from the Tehran University of Medical Sciences Research Ethics Board (IR.TUMS.FNM.REC.1403.170) on December 23, 2024. The study was conducted in accordance with the Declaration of Helsinki. Permission to translate and use the Palliative Nursing Care Quality Scale (PNCQS) was granted by the original scale developer.

Participation was entirely voluntary and anonymous; no identifying information was collected from participants. No financial or non-financial incentives were provided. To mitigate potential coercion in the hierarchical hospital environment, an independent researcher (not employed in the participants’ hospitals) approached eligible nurses, explained the study objectives and procedures, and explicitly emphasized that participation or withdrawal would have no impact on their employment, performance evaluation, or professional relationships. Participants were assured that all data would be kept confidential and used solely for research purposes. Written informed consent was obtained from all participants prior to data collection.

### 2.2 Assessment of psychometric properties

#### 2.2.1 Phase 1: Translation Process.

To evaluate the psychometric properties of the scale, the translation procedure followed the World Health Organization (WHO) guidelines [28]. Initially, three translators proficient in both Persian and English with expertise in medical terminology independently translated the original questionnaire into Persian. A consensus meeting was then arranged, during which the translators compared their translations, resolved inconsistencies, and selected the most appropriate wording for each item. Subsequently, two other bilingual translators, who had no knowledge of the original scale or the research objectives, back‑translated the finalized Persian version into English. The research team compared this back‑translated version with the original scale. Finally, the back‑translated version was submitted to the original instrument developer (Zulueta Egea) for approval, leading to the final Persian version of the questionnaire.

#### 2.2.2 Phase 2: Instrument Validation.

In line with the COSMIN guidelines for outcome measurement instruments [16], the psychometric assessment of the PNCQS covered three validity types (face, content, and construct) and two reliability aspects (internal consistency and test‑retest).

**Face Validity**: A mixed‑methods (qualitative‑quantitative) approach was adopted. Quantitatively, 15 eligible nurses rated each item’s clarity on a 5‑point Likert scale (1 = very difficult to understand, 5 = very easy to understand). The impact score for each item was derived using the formula: Impact Score = Frequency (%) × Comprehensiveness [29]. An impact score above 1.5 was deemed acceptable. Qualitatively, the same 15 nurses participated in in‑depth face‑to‑face interviews where they discussed each item’s difficulty, relevance, ambiguity, and provided paraphrasing. Based on their feedback, the research team reviewed and iteratively improved the items.

**Content Validity:** A similar qualitative‑quantitative approach was used. For the qualitative part, a purposive panel of ten experts—comprising four nursing faculty, two oncologists, and four experienced nurses—assessed the questionnaire for grammatical accuracy, wording suitability, item ordering, and scoring method.

For the quantitative assessment, the Content Validity Ratio (CVR) and Content Validity Index (CVI) were applied [19]. To compute CVR, experts rated each item on a three‑point scale (essential, useful but not essential, not essential). With ten experts, a CVR > 0.62 was considered acceptable [30]. For CVI, experts rated item relevance on a four‑point scale (1 = not relevant, 4 = highly relevant). The item‑level CVI (I‑CVI) was calculated as the proportion of experts rating an item 3 or 4. The scale‑level CVI (S‑CVI) was the mean of all I‑CVI scores. I‑CVI above 0.79 [31] and S‑CVI above 0.90 were considered satisfactory [19].

**Construct Validity:** Construct validity was examined in two sequential steps

#### Step 1 – Testing the original one‑factor model.

A confirmatory factor analysis (CFA) was first run on the original 20‑item unidimensional structure proposed by Zulueta Egea et al. (2020), using a sample of 220 nurses. This step was essential to see if the original factor structure could be directly transferred to the Iranian context.

#### Step 2 – Exploratory factor analysis (EFA) and subsequent CFA.

Given that the original one‑factor model was anticipated to have poor fit (based on initial analyses), we performed EFA to uncover the factor structure in this population. The same 220 participants were used for EFA. Sampling adequacy was checked with the Kaiser‑Meyer‑Olkin (KMO) measure and Bartlett’s test of sphericity. Factor extraction was performed using principal axis factoring with Direct Oblimin rotation (Kaiser normalization). The number of factors to retain was initially determined using Kaiser’s eigenvalue-greater-than-one criterion and the scree plot [32].

In addition, parallel analysis was conducted in RStudio using the fa.parallel function from the psych package to further evaluate and confirm the optimal number of factors to retain. Items with factor loadings below 0.40 or low communalities (<0.30) were candidates for removal.

After EFA revealed a four‑factor solution (Family‑Centered Care, Empathic and Spiritual Care, Hope and Life Review, Physical Symptom Management), a second CFA was conducted on an independent sample of 400 nurses to validate the extracted model. Model fit was evaluated using χ²/df, GFI, AGFI, CFI, TLI, RMSEA, NFI, IFI, PNFI, and PCFI [33]. Standardized factor loadings of at least 0.40 were considered acceptable.

**Preliminary data checks:** Before factor analyses, data normality was assessed using skewness (within ±3), kurtosis (within ±7), and the Kolmogorov–Smirnov test. The item distributions demonstrated acceptable univariate normality, and the questionnaire employed a five-point Likert response scale. Given these characteristics, the ordinal responses were treated as approximately continuous, and confirmatory factor analysis was performed in AMOS using maximum likelihood (ML) estimation with bootstrapping (1,000 resamples) to obtain robust parameter estimates and confidence intervals. Although weighted least squares estimation is often recommended for ordinal data, ML estimation is widely used and considered acceptable for five-category Likert-type items when the sample size is adequate and the distributional assumptions are reasonably satisfied. The psychometric evaluation was conducted in accordance with COSMIN recommendations [34,35].

**Convergent and Discriminant Validity:** The evaluation of convergent validity followed the procedure proposed by Fornell and Larcker (1981) [36], which involved computing the Average Variance Extracted (AVE) and Composite Reliability (CR) for each construct. AVE values exceeding 0.50 and CR values above 0.70 were regarded as satisfactory. To assess discriminant validity, the Maximum Shared Squared Variance (MSV) was compared against the AVE for each factor; discriminant validity was established when the MSV was lower than the corresponding AVE. All statistical analyses were carried out using SPSS version 24 and AMOS version 24. There were no missing data in the dataset, as all questionnaires were completely filled out under close supervision, strictly adhering to the predefined inclusion and exclusion criteria. It is worth noting that criterion validity was not evaluated because no validated Persian gold-standard instrument is currently available for assessing the quality of palliative nursing care. Consequently, criterion-related validity could not be examined in the present study.

**Reliability:** The reliability of the scale was examined through both internal consistency and temporal stability. Internal consistency was determined using Cronbach’s alpha (α) for the total scale and its subscales. Values of 0.70 or higher for both coefficients were considered acceptable [37]. For temporal stability, a test‑retest approach was adopted. A distinct subsample of 30 nurses from the main study population completed the PNCQS a second time after an interval of two weeks. This interval was selected because the attribute of palliative nursing care quality is not anticipated to change substantially over such a brief period in the absence of any intervention. Test‑retest reliability was quantified using the Intraclass Correlation Coefficient (ICC) with a 95% confidence interval. ICC values greater than 0.75 were deemed acceptable [38], consistent with the recommendations of the COSMIN guideline.

## 3. Results

### 3.1 Participant characteristics

The study included 620 participants. The majority were female (87.2%) and married (51.8%). Regarding educational attainment, most participants held a bachelor’s degree (83.9%), followed by master’s degree (13.2%) and PhD (2.4%). In terms of city of service, half of the participants worked in Tehran (50.0%), 30.0% in Tabriz, and 20.0% in Ardabil. The vast majority served in intensive care units (91.1%), with the remaining 8.9% working in oncology units. Concerning employment status, 65.7% had permanent contracts and 33.8% had temporary contracts. Shift patterns were predominantly rotating (72.1%), while 16.7% worked morning shifts, 7.5% afternoon shifts, and 3.2%-night shifts. The mean age of participants was 34.77 years (SD = 7.94), and the mean work experience was 10.58 years (SD = 7.78) (Table 1).

**Table 1:** Demographic characteristics of participants.

Frequency (percentage)		Demographic characteristics
gender	female	543 (87.58)
	male	77 (12.42)
Marital status	single	297 (47.90)
	married	323 (52.10)
Educational status	Bachelor degree	523 (84.40)
	Master degree	82 (13.20)
	PhD	15 (2.40)
City of service	Tehran	310 (50.00)
	Tabriz	186 (30.00)
	Ardabil	124 (20.00)
ward of service	ICU	564 (91.10)
	Oncology	56 (8.90)
Type of contract	Permanent	409 (66.0)
	Temporary	211 (34.00)
shift	Morning	104 (16.80)
	Afternoon	47 (7.60)
	Night	20 (3.20)
	Rotating	449 (72.40)
Age (Mean ± SD)	34.77 ± 7.94 years	
work experience (Mean ± SD)	10.58 ± 7.78 years	

### 3.2 Face validity

The item impact scores for all items ranged from 1.51 to 3.6, with every item exceeding the threshold of 1.5. A detailed summary of item-level impact scores is provided in S1 Table. This finding indicates that the target population understood the items adequately. Accordingly, all items were deemed appropriate and none were removed. In addition, a qualitative assessment of face validity was carried out to further improve item clarity. The qualitative results revealed that the items effectively communicated the intended concepts, and no modifications were required at this stage.

### 3.3 Content validity

The Content Validity Ratio (CVR) for the scale items ranged from 0.8 to 1.0, and the Item‑level Content Validity Index (I‑CVI) ranged from 0.9 to 1.0. According to Lawshe’s table, items with a CVR below 0.62 are considered unnecessary, and those with an I‑CVI below 0.79 are regarded as inappropriate. All 20 items of the Persian version exceeded these criteria and were therefore retained. The overall Scale‑level Content Validity Index (S‑CVI) was 0.99, reflecting excellent content validity.

### 3.4 Construct validity

#### Step 1 – Testing the original unidimensional model (20 items).

Before performing exploratory factor analysis (EFA), we initially evaluated the original one-factor structure proposed by Zulueta Egea et al. (2020) by applying confirmatory factor analysis (CFA) to the Persian translation. The model demonstrated inadequate fit across all indices (see Table 2). This outcome confirmed that the original single-factor model was unsuitable for the Iranian context, consequently justifying an exploratory approach.

**Table 2:** Fit indices for the original 20-item unidimensional CFA model.

Indicator	Model estimate	Acceptable limit	Result
χ²/df	3.19	≤ 3	Not approved
NFI	0.74	≥ 0.90	Not approved
CFI	0.80	≥ 0.90	Not approved
TLI	0.78	≥ 0.90	Not approved
IFI	0.80	≥ 0.90	Not approved
GFI	0.79	≥ 0.90	Not approved
PNFI	0.68	≥ 0.50	Approved
PCFI	0.78	≥ 0.50	Approved
RMSEA	0.10	≤ 0.08	Not approved

**Note:** χ²/df: Chi-Square to Degrees of Freedom Ratio; NFI: Normed Fit Index; CFI: Comparative Fit Index; TLI: Tucker-Lewis Index; IFI: Incremental Fit Index; GFI: Goodness of Fit Index; PNFI: Parsimonious Normed Fit Index; PCFI: Parsimony Comparative Fit Index; RMSEA: Root Mean Square Error of Approximation.

#### Step 2 – Exploratory Factor Analysis (EFA).

The Kaiser-Meyer-Olkin (KMO) measure of sampling adequacy was 0.916, and Bartlett’s test of sphericity yielded a significant result (χ² = 2131.889, df = 190, p < 0.001), indicating that the data were appropriate for factor analysis.

Using principal axis factoring with Direct Oblimin rotation, four factors with eigenvalues greater than 1 were extracted (initial eigenvalues: 8.531, 1.343, 1.232, and 1.057), accounting for 51.41% of the extracted common variance. Inspection of the scree plot showed a clear inflection after the fourth factor (Fig 1), and parallel analysis likewise supported the retention of a four-factor solution (Fig 2).

**Fig 1:**
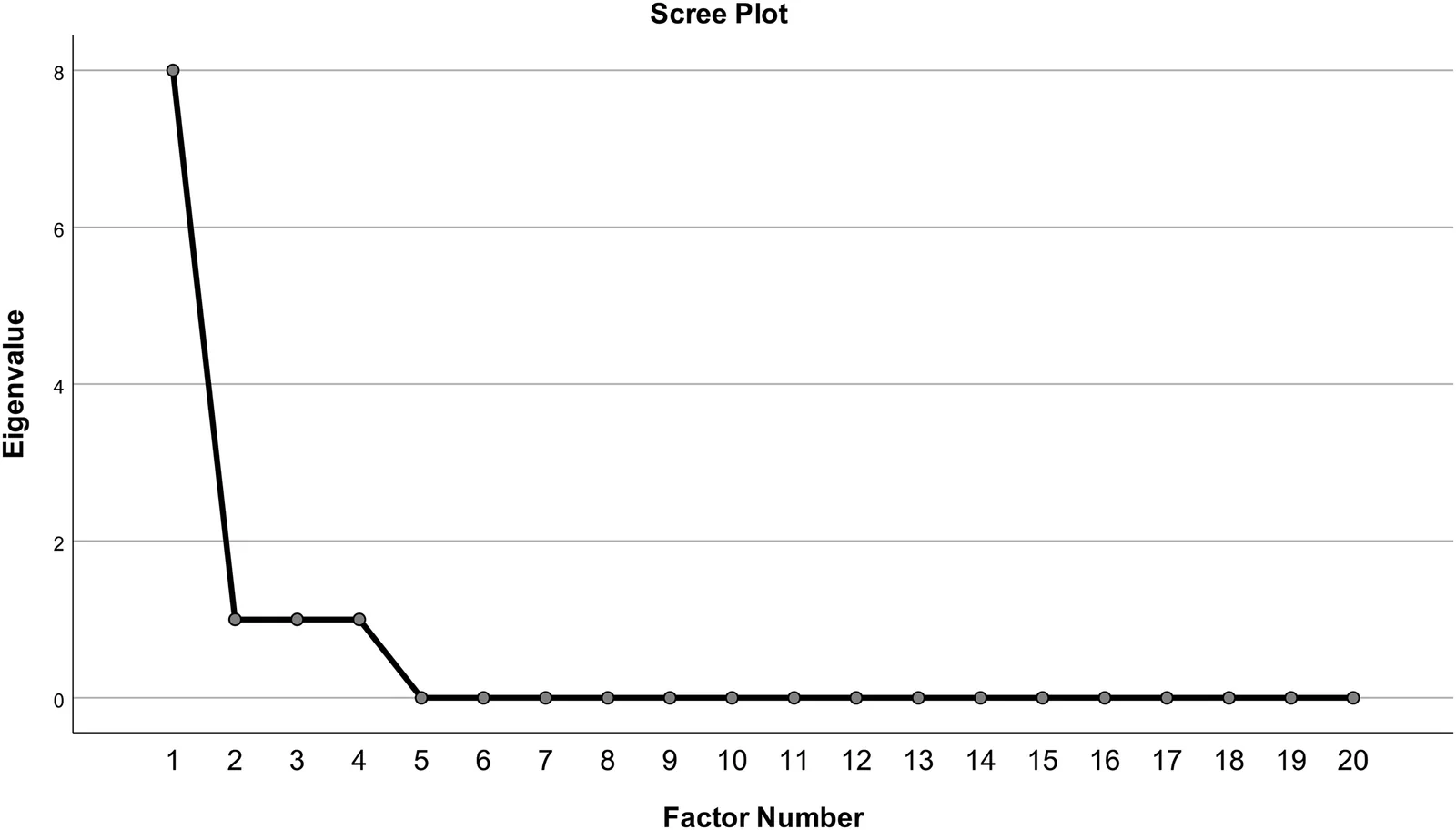
Scree plot of eigenvalues for the Persian version of the PNCQS.

**Fig 2:**
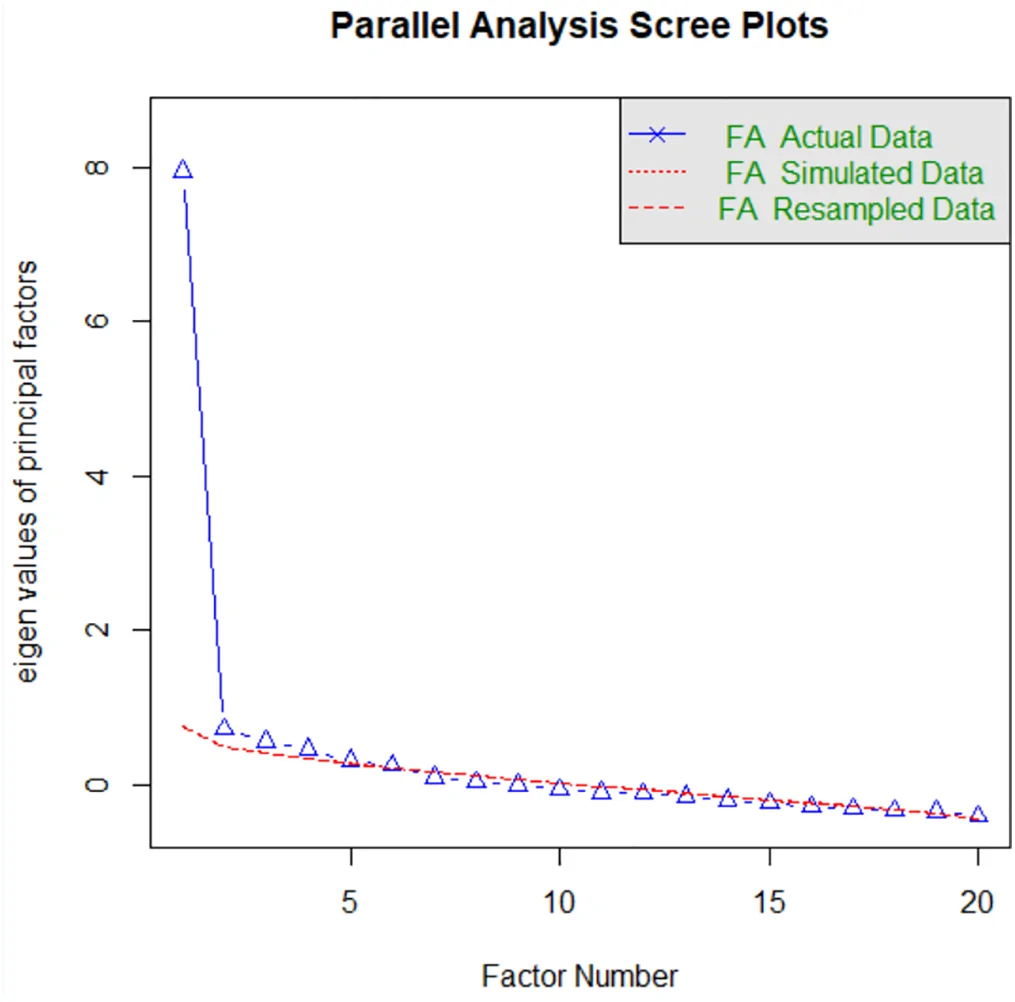
Parallel analysis for factor retention in the Persian version of the PNCQS.

The Pattern Matrix loadings, representing the unique contributions of each item, are reported in Table 3. The primary factor loadings ranged from 0.419 to 0.753, with all retained items loading highest on their intended factors. The convergence of evidence from the Kaiser criterion (eigenvalues >1), the scree plot (Fig 1), and parallel analysis (Fig 2) provided strong empirical support for the four-factor solution. The extracted factors were labeled as follows: Family-Centered Care (6 items), Empathic and Spiritual Care (7 items), Hope and Life Review (5 items), and Physical Symptom Management (2 items).

**Table 3:** Pattern Matrix Loadings and Communalities from EFA with Oblimin Rotation (N = 220).

Latent factor	Item	Factor 1	Factor 2	Factor 3	Factor 4	Communalities	Eigenvalue	% of variance
Family-Centered Care	3	.49				.44	8.53	15.02
	4	.52				.52		
	5	.75				.64		
	6	.66				.54		
	7	.45				.51		
	8	.51				.58		
Empathic and Spiritual Care	10		.44			.48	1.34	13.70
	11		.74			.60		
	12		.59			.60		
	13		.47			.41		
	14		.44			0.50		
	19		.43			.42		
	20		.42			.40		
Hope and Life Review	9			.49		.40	1.23	12.75
	15			.56		.57		
	16			.52		.54		
	17			.74		.59		
	18			.57		.50		
Physical Symptom Management	1				.67	.50	1.05	9.94
	2				.70	.55		
Total	–	–	–	–	–	–	–	51.41

Although four items on the Empathic and Spiritual Care factor (q10, q14, q19, and q20) showed loadings between 0.42 and 0.44—just above the 0.40 cutoff—they were theoretically essential for capturing the Iranian cultural and spiritual nuances of palliative care. Their retention is consistent with standard cross-cultural validation practices [26,32].

#### Step 3 – Confirmatory Factor Analysis (CFA) on the four‑factor model.

A confirmatory factor analysis was performed on the four‑factor, 20‑item model using an independent sample of 400 nurses. The CFA model is presented in Fig 3. All items demonstrated standardized factor loadings exceeding 0.40 (p < 0.001). As presented in Table 4, the model showed excellent fit across all indices. Compared to the original one‑factor model, the four‑factor model exhibited considerably better fit (see Table 2 versus Table 4). All standardized factor loadings were above 0.40 (p < 0.001). Full CFA parameter estimates, including standardized factor loadings, error variances, and correlations between latent factors, are presented in S2 Table.

**Table 4:** Fit indices for the final 20‑item four‑factor CFA model.

Indicator	Model estimates	Permissible limit	Result
χ2/df	2.84	≤ 3	Approved
NFI	.93	≥ 0.90	Approved
CFI	.95	≥ 0.90	Approved
TLI	.95	≥ 0.90	Approved
IFI	.95	≥ 0.90	Approved
GFI	.90	≥ 0.90	Approved
PNFI	.80	≥ 0.50	Approved
PCFI	.82	≥ 0.50	Approved
RMSEA	.06	≤ 0.08	Approved

**Note:** χ²/df: Chi-Square to Degrees of Freedom Ratio; NFI: Normed Fit Index; CFI: Comparative Fit Index; TLI: Tucker-Lewis Index; IFI: Incremental Fit Index; GFI: goodness of fit index; PNFI: Parsimonious Normed Fit Index; PCFI: Parsimony Comparative Fit Index; RMSEA: Root Mean Square Error of Approximation

**Fig 3:**
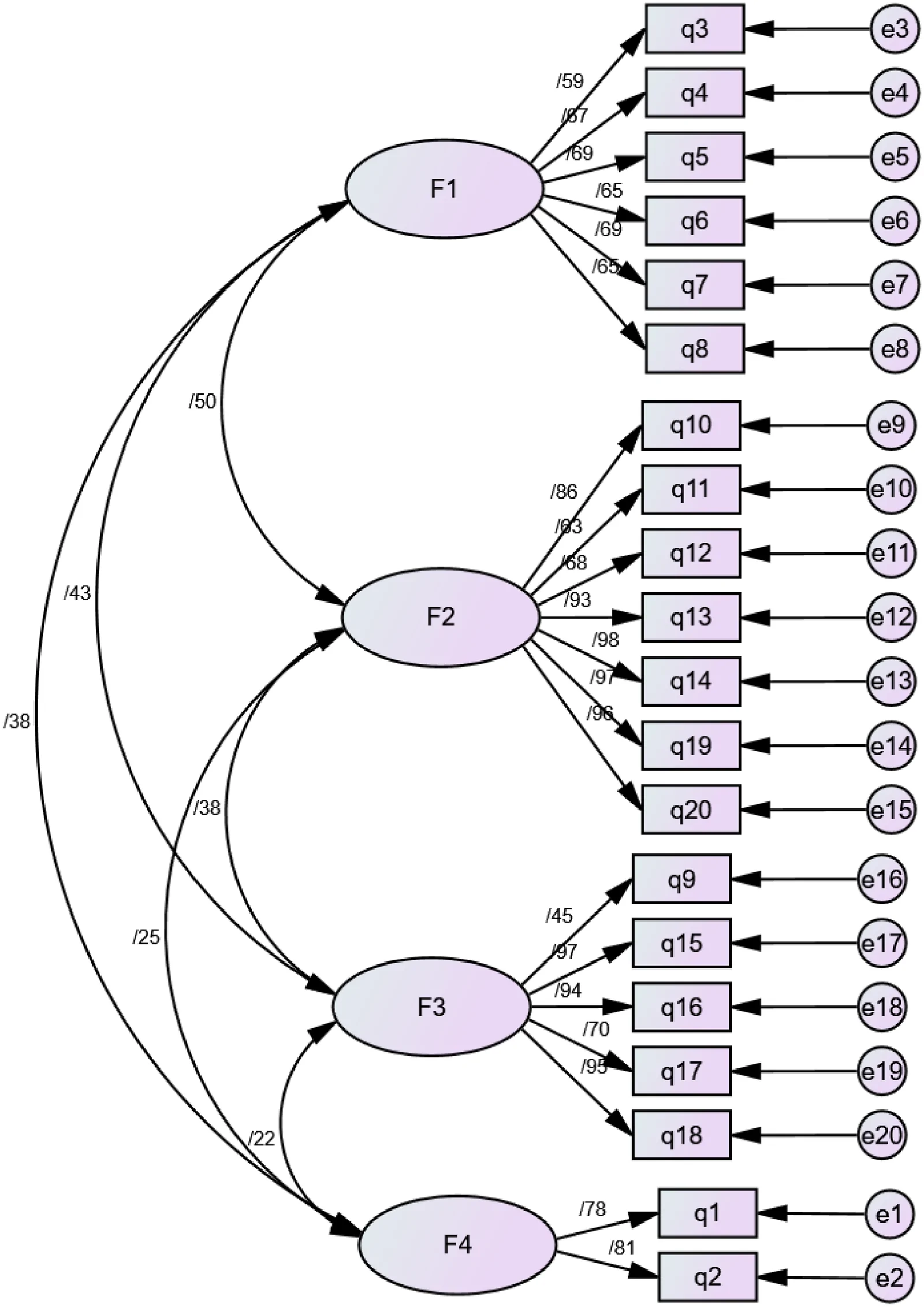
Confirmatory factor analysis (CFA) model of the Persian version of the PNCQS (n = 400). Standardized factor loadings, measurement error terms, and correlations among the four latent factors are shown. F1 = Family-Centered Care; F2 = Empathic and Spiritual Care; F3 = Hope and Life Review; F4 = Physical Symptom Management.

### 3.5 Convergent and discriminant validity, construct reliability

Following the Fornell‑Larcker approach, we computed the composite reliability (CR), average variance extracted (AVE), and maximum shared squared variance (MSV) for each factor. As shown in Table 5, CR values across the four factors ranged from 0.77 to 0.95, all above the recommended cutoff of 0.7. The AVE values were 0.42 for Family‑Centered Care, 0.75 for Empathic and Spiritual Care, 0.68 for Hope and Life Review, and 0.62 for Physical Symptom Management. Although the AVE for the Family‑Centered Care factor was below the conventional threshold of 0.50, the composite reliability for this factor was acceptable (CR = 0.81). All standardized factor loadings for this factor in the CFA were significant and ranged from 0.52 to 0.78 (p < 0.001), indicating that the low AVE was not due to weak or problematic items. For all factors, the MSV values were lower than their corresponding AVE values, with a range of 0.14 to 0.25.

**Table 5:** Convergent and discriminant validity indices.

Factor	CR	AVE	MSV
Family-Centered Care	0.81	0.42	0.25
Empathic and Spiritual Care	0.95	0.75	0.25
Hope and Life Review	0.91	0.68	0.18
Physical Symptom Management	0.77	0.62	0.14

CR: Composite Reliability; AVE: Average Variance Extracted; MSV: Maximum Shared Squared Variance.

### 3.6. Reliability

The internal consistency of the scale was assessed using Cronbach’s alpha. The Cronbach’s alpha values for the subscales and the total scale ranged from 0.77 to 0.83. For the two-item Physical Symptom Management subscale, due to the known instability of Cronbach’s alpha with very short scales, the inter-item correlation (r = 0.77) and the Spearman-Brown coefficient (0.77) were also calculated. The test‑retest method was used to evaluate the scale’s stability, yielding an ICC of 0.76 for the total instrument (Table 6).

**Table 6:** Cronbach’s alpha coefficient for the Four-factor model, 20 items (n = 620).

Factor	Number of items	Cronbach’s alpha	ICC (Average Measures)	Spearman-Brown Coefficient
Family-Centered Care	6	0.83		
Empathic and Spiritual Care	7	0.83		
Hope and Life Review	5	0.81		
Physical Symptom Management	2	0.76	0.77	0.77
Total	20	0.92		
	

## 4. Discussion

This study established the Persian version of the Palliative Nursing Care Quality Scale (PNCQS) as a reliable and valid instrument for assessing the quality of palliative nursing care among Iranian nurses. A major strength of the study was the sequential psychometric validation strategy, in which the original one-factor structure was first tested using confirmatory factor analysis, followed by exploratory factor analysis after the original model failed to demonstrate acceptable fit, and finally cross-validated through confirmatory factor analysis in an independent sample. This rigorous approach strengthens the credibility and stability of the proposed four-factor structure. The final model demonstrated satisfactory construct validity, discriminant validity, internal consistency, and temporal stability, supporting the Persian PNCQS as a culturally appropriate instrument for clinical practice, research, and quality improvement in Iranian palliative care settings.

The socio‑ecological framework [39], which emphasizes interactions between the individual, environment, culture, and social structures, provides a suitable conceptual lens for analyzing the experiences of Iranian nurses.

A key strength of the study lies in its strict adherence to the COSMIN guidelines, which ensured a systematic and comprehensive evaluation of all relevant psychometric properties [16]. Furthermore, the large and socio‑demographically diverse sample enhances the generalizability of the results across urban Iranian hospital populations. An additional methodological strength was the use of independent samples for exploratory and confirmatory factor analyses, thereby minimizing the risk of overfitting and providing stronger evidence for the stability and reproducibility of the final measurement model.

A notable finding is the shift from the original unidimensional structure [14] to a culturally meaningful four-factor structure in the present Iranian sample. The initial confirmatory factor analysis demonstrated that the original one-factor model did not adequately fit the Iranian data, indicating that the latent structure proposed in the original validation could not be replicated directly in this context. Consequently, exploratory factor analysis was undertaken to identify the underlying construct, and the resulting four-factor solution was subsequently confirmed in an independent sample through confirmatory factor analysis. The four extracted factors—Family‑Centered Care, Empathic and Spiritual Care, Hope and Life Review, and Physical Symptom Management—explained a substantial proportion of the total variance.

Notably, the Physical Symptom Management factor consisted of two items assessing pain control and overall symptom relief. Although this limited number of items may not fully capture the breadth of physical symptom management, both items demonstrated satisfactory factor loadings and acceptable internal consistency (α = 0.76). Furthermore, the inter-item correlation and Spearman-Brown coefficient supported the reliability of this two-item subscale, complementing the Cronbach’s alpha findings. This is consistent with previous studies showing that well‑designed two‑item subscales can demonstrate adequate reliability when supplemented with Spearman‑Brown correction [40,41], supporting their retention as a core dimension of palliative nursing care quality. This multidimensional structure aligns with the holistic nature of palliative care, which encompasses physical, psychological, social, and spiritual domains [42].

In contrast, the Chinese version of the PNCQS validated by Liu et al. [24] retained a unidimensional structure. The difference between the Chinese and Persian versions is likely attributable not only to cultural variations but also to differences in psychometric evaluation strategies. While the Chinese validation confirmed the original model, the present study first empirically tested the original one-factor structure and only proceeded to exploratory analysis after demonstrating inadequate model fit. This discrepancy highlights the profound influence of cultural factors on how nurses perceive palliative care quality. In a collectivist culture with strong Islamic values, dimensions such as “family” and “spirituality” emerge as distinct from other aspects.

In Iranian culture, extensive family involvement, spiritual support, and hope‑giving are highly valued. Palliative care in Iran is often delivered at home with the family as the core provider, and most care‑related burdens are shouldered by the family. Evidence indicates that family‑centered empowerment programs significantly improve the quality of life of family caregivers. In Iranian society, the synergy between an active family role and the support of religious‑spiritual values creates a unique phenomenon in end‑of‑life care that has no direct equivalent in Western models. Research in Iran has highlighted persistent unmet spiritual needs related to meaning, hope, and social support [43]. Our findings, which show the convergence of different care dimensions, are consistent with other studies from Turkey [44] and Arab countries [45], underscoring the need to align assessment tools with cultural specificities.

Convergent validity, assessed using the Fornell‑Larcker criterion, showed that composite reliability values for all four factors exceeded the recommended threshold, indicating good internal consistency. The average variance extracted values for three factors (Empathic and Spiritual Care, Hope and Life Review, and Physical Symptom Management) were above the acceptable level, confirming adequate convergent validity for these subscales. However, the AVE for Family‑Centered Care fell below the conventional threshold. According to Fornell and Larcker (1981) [36], an AVE below the recommended value does not automatically invalidate a construct provided that composite reliability is adequate and all item loadings are significant. The acceptable composite reliability for this factor and the significance of its factor loadings supports its retention. Moreover, the successful replication of the four-factor solution in the independent CFA sample further supports the structural validity of this construct despite its relatively lower AVE.

Nevertheless, the suboptimal AVE for Family‑Centered Care warrants deeper consideration, particularly in light of the cultural complexity and heterogeneity inherent in the Iranian context. In collectivist and family‑oriented societies like Iran, Family‑Centered Care extends beyond structured clinical activities to encompass fluid, emotionally charged, and culturally embedded practices, including shared decision‑making, emotional containment, advocacy, and indirect spiritual support [22,43]. This broad and multifaceted nature may generate greater response variability among nurses, as their perceptions of family involvement are shaped by individual values, familial roles, religious beliefs, and the specific dynamics of each patient‑family unit. Such heterogeneity is not a methodological flaw but rather a reflection of the construct’s rich and context‑dependent manifestation in real‑world palliative care settings. Moreover, the items within this factor may tap into both observable behaviors (e.g., communication with family) and more subjective attitudes (e.g., respect for family values), which could further attenuate the shared variance captured by AVE. Nevertheless, this explanation does not diminish the factor’s conceptual relevance; rather, it underscores the need to interpret the AVE value with caution while acknowledging the multifaceted nature of Family‑Centered Care in this cultural context.

Discriminant validity was fully established for all four factors, indicating that the four dimensions measure distinct yet related aspects of palliative nursing care quality. Together with the satisfactory CFA fit indices obtained in the validation sample, these findings provide robust evidence that the Persian PNCQS possesses a stable multidimensional measurement structure. These convergent and discriminant validity findings are comparable to those reported in studies measuring cultural competence among Iranian nurses, further strengthening the explanatory power of the dimensions.

Internal consistency was excellent for the total scale and acceptable for all subscales. For the two-item Physical Symptom Management subscale, the Cronbach’s alpha value remained within the acceptable range, a finding justified by the subscale’s brevity. Although Cronbach’s alpha is known to be sensitive to the number of items in a scale [46], and may underestimate true reliability for two-item measures [40], the obtained value meets the generally accepted threshold of ≥0.70 for research purposes [47]. Numerous studies in the nursing and healthcare literature have likewise reported acceptable Cronbach’s alpha coefficients for two-item subscales, reinforcing that internal consistency is not necessarily compromised by using a small number of theoretically sound items [41]. In addition, the satisfactory composite reliability coefficients observed across all factors further support the internal consistency of the instrument. Test-retest reliability over a two-week interval indicated moderate temporal stability, which is considered acceptable for a new instrument in a dynamic clinical setting.

The Chinese validation of the PNCQS [24] retained the original unidimensional structure with a cumulative variance contribution rate of 60.03% and a total Cronbach’s α of 0.96, demonstrating that even among non‑Western countries, considerable cross‑cultural differences exist. Iran’s collectivist culture with its emphasis on extended family and spiritual values operates differently from China’s relatively state‑focused social structure. Numerous other studies in the Middle East, while emphasizing the separation between nurses’ perceptions and actions in family‑centered care, have confirmed the stronger role of spirituality and family support in Islamic cultural contexts. The World Health Organization (WHO) defines palliative care as an approach that aims to improve the quality of life of patients and families through prevention, relief of suffering, early identification, and treatment of pain and other symptoms across physical, psychosocial, and spiritual domains [48], a definition that strongly supports the multidimensional framework identified in our study. Overall, these findings suggest that although the core concept of palliative nursing care quality is universal, its latent dimensions are influenced by cultural context, highlighting the importance of validating measurement instruments rather than assuming cross-cultural equivalence of the original factor structure.

### Implications for practice and research

The Persian PNCQS provides a standardized, psychometrically robust instrument for Iranian health managers, policy-makers, clinicians, and researchers to evaluate the quality of palliative nursing care. The rigorously validated four-factor structure enables comprehensive assessment of key dimensions of palliative care, facilitating targeted quality improvement initiatives. For example, low scores on Family-Centered Care may guide interventions to strengthen family engagement, whereas deficiencies in Empathic and Spiritual Care may indicate the need for education and training in communication and spiritual support. Moreover, the availability of a culturally adapted multidimensional instrument enables researchers to investigate the relationships between different domains of palliative nursing care and important patient and family outcomes, including symptom burden, quality of life, family satisfaction, and care experiences. The robust cross-validation of the factor structure also provides a sound foundation for future longitudinal studies, intervention evaluations, and cross-cultural comparisons of palliative nursing care quality.

### Limitations and future directions

This study has several limitations. The use of convenience sampling at the ward level may have introduced selection bias, despite the large sample size. Self‑reported data are susceptible to social desirability bias. Furthermore, we did not conduct multigroup confirmatory factor analysis (MGCFA) to examine measurement invariance across key demographic subgroups (e.g., gender, ICU vs. Oncology units, or cities). Consequently, the stability of the factor structure across these subgroups could not be established. Such analyses would provide stronger evidence for the scale’s generalizability and comparability across diverse populations. We acknowledge this as a limitation and recommend that future studies with larger and more balanced samples perform MGCFA to evaluate configural, metric, and scalar invariance across relevant subgroups, thereby providing additional evidence for the scale’s measurement invariance and cross-population applicability. The predominantly female sample (87.2%) working in intensive care units (91.1%) limits generalizability to male nurses and other clinical settings. The Physical Symptom Management subscale contained only two items, which may restrict content coverage; however, as discussed earlier, the inter‑item correlation and Spearman‑Brown coefficient indicated acceptable reliability for this brief subscale.

The AVE for Family‑Centered Care was below the recommended threshold (0.42). As discussed earlier, this may reflect cultural complexity and heterogeneity in Family‑Centered Care practices within the Iranian context, rather than a fundamental structural flaw. Nevertheless, this finding warrants further investigation.

Future research should implement random sampling at all stages to enhance generalizability, assess criterion validity against other palliative care measures, and evaluate the scale’s responsiveness to educational or policy interventions. Additionally, given the cultural uniqueness of palliative care in Iran, future studies may consider refining or expanding the Family‑Centered Care and Physical Symptom Management subscales—through qualitative inquiry and item development—to enhance their psychometric robustness and cultural relevance. However, such refinements should be undertaken with caution to preserve the theoretical integrity and practical utility of the existing scale.

## 5. Conclusion

The Persian version of the PNCQS is a valid, reliable, and culturally appropriate instrument for assessing the quality of palliative nursing care among Iranian nurses. The sequential psychometric validation process, including initial testing of the original model, exploratory factor analysis, and cross-validation through confirmatory factor analysis in an independent sample, provides strong evidence for the stability and robustness of the proposed four-factor structure. Although the Family-Centered Care subscale demonstrated a slightly lower average variance extracted than the recommended threshold, its acceptable composite reliability, significant factor loadings, and successful replication in the independent validation sample support its retention. Overall, the Persian PNCQS fills an important gap in palliative care assessment in Iran and provides a robust multidimensional instrument for clinical practice, quality improvement, education, and research. Future studies may further refine the Family-Centered Care subscale and evaluate the scale in other healthcare settings and populations.

## Supporting information

S1 Questionnaire Persian version of the Palliative Nursing Care Quality Scale (PNCQS). (PDF)

S1 Table Impact Scores of the PNCQS Items. (DOCX)

S2 Table Standardized Factor Loadings, Error Variances, and Latent Factor Correlations. (DOCX)

## Abbreviations

PNCQS: Palliative Nursing Care Quality Scale
POS: Palliative Outcome Scale
CFA: Confirmatory factor analysis
WHO: World Health Organization’s
SEM: Structural Equation Modeling
IS: Impact Score
CVR: Content Validity Ratio
CVI: Content Validity Index
I-CVI: Item-Content Validity Index
S-CVI: Scale-Content Validity Index
EFA: Exploratory Factor Analysis
KMO: Kaiser-Meyer-Olkin
GFI: Goodness-Of-Fit Index
AGFI: Adjusted Goodness-Of-Fit Index
RMSEA: Root Mean Square Error of Approximation
CFI: Comparative Fit Index
ICC: Intraclass Correlation Coefficient
CR: Composite Reliability
AVE: Average Variance Extracted
MSV: Maximum Shared Squared Variance
