# Supplementary material for: Psychometric evaluation of the Palliative Nursing Care Quality Scale (PNCQS) among ICU and oncology nurses in Iran
Source: PLoS One. 2026 Nov 4;21(11):e0361384. doi: 10.1371/journal.pone.0361384 (PMC13734446; doi:10.1371/journal.pone.0361384)
Supplement: Supplementary file 1 [file pone.0361384.s001.pdf]

به نام خدا

با سلام و احترام

این پرسشنامه برای انجام طرح تحقیقاتی با عنوان " بررسی ویژگی های روانسنجی مقیاس کیفیت مراقبت پرستاری تسکینی (PNCQS) در پرستاران " در اختیار شما قرار گرفته است. خواهشمند است در صورت رضایت برای تکمیل این پرسشنامه با جواب‌های صادقانه خویش پژوهشگران را در انجام این پروژه یاری فرمائید. در ضمن یادآوری می‌شود نیازی به ذکر نام و نام خانوادگی در پرسشنامه نیست و تمامی اطلاعات ارائه شده توسط شما محرمانه بوده و به‌صورت کلی تحلیل خواهد شد. از عنایت، دقت نظر و بردباری شما در تکمیل این پرسشنامه بسیار سپاسگذاریم.

مجری طرح: الهه حبیب پور

الف) مشخصات فردی اجتماعی

۱- سن...........

۲- جنس: زن o مرد o

۳- وضعیت تاهل: مجرد o متاهل o مطلقه o بیوهo

۴- میزان تحصیلات: لیسانس o فوق لیسانس o دکتری o

۵- وضعیت اشتغال: قراردادیo طرحی o تمدید طرح o پیمانی o رسمی o

۶- نوع شیفت: صبح o عصر o شب o چرخشیo

۷- نوع بیماران مراقبت شونده: بزرگسال o نوزاد o کودکo

۸- سابقه کار: ....

۹- نام بخش: ....

پرسشنامه PNCQS					
	هرگز	به‌ندرت	گاهی‌اوقات	اغلب	همیشه
۱. من ارزیابی جامعی از درد انجام می‌دهم که شامل عواملی مانند محل، فراوانی، شدت و تأثیر آن بر کیفیت زندگی بیمار است.	☐	☐	☐	☐	☐
۲. من اقدام‌های لازم برای مدیریت علائم (اضطراب، ضعف، سردرگمی، تنگی نفس، درد، یبوست، خشکی دهان و استفراغ) را فراهم می‌کنم.	☐	☐	☐	☐	☐
۳. . من با پرسیدن از بیماران و/یا اعضای خانواده درباره رفاه یا راحتی آن‌ها، اثربخشی مراقبت را ارزیابی می‌کنم.	☐	☐	☐	☐	☐
۴. من ارتباط با اعضای خانواده و/یا مراقبین اصلی را در یک محیط آرام و خصوصی تشویق می‌کنم.	☐	☐	☐	☐	☐
۵. . من اعضای خانواده و/یا مراقبین اصلی را در مراقبت از بیماران به‌طور برنامه‌ریزی‌شده بر اساس ویژگی‌های آن‌ها درگیر می‌کنم.	☐	☐	☐	☐	☐
۶. من ارزیابی می‌کنم که آیا خانواده و/یا مراقب اصلی تحت فشار بیش از حدی از مراقبت قرار دارند یا خیر.	☐	☐	☐	☐	☐
۷. من از اعضای خانواده و/یا مراقبین اصلی در مراقبت و همراهی بیماران حمایت می‌کنم.	☐	☐	☐	☐	☐
۸. من اعضای خانواده و/یا مراقبین اصلی را تشویق می‌کنم تا احساسات خود را درباره فقدان و غم بیان کنند.	☐	☐	☐	☐	☐
۹. . من درک می‌کنم که بیماران و/یا خانواده‌ها چه نشانه‌های غیرکلامی را منتقل می‌کنند و کلمات خاصی را که می‌خواهند از آن‌ها اجتناب کنند.	☐	☐	☐	☐	☐
۱۰. من می‌دانم چگونه به‌طور فعال در گوش دادن مؤثر شرکت کنم و احساسات، افکار و اعتقادات بیماران و/یا خانواده‌ها را درک کنم، در حالی که از قضاوت کردن خودداری می‌کنم.	☐	☐	☐	☐	☐
۱۱. من در زمانی که بیماران و/یا اعضای خانواده احساساتی مانند ترس، خشم یا غم را ابراز می‌کنند، درک و گرمی خود را به آن‌ها منتقل می‌کنم.	☐	☐	☐	☐	☐
۱۲. من به بیمار و/یا اعضای خانواده کمک می‌کنم تا در مورد نگرانی‌های مربوط به مسائل معلق، تأمل یا روشن‌سازی کنند.	☐	☐	☐	☐	☐
۱۳. من در دشوارترین زمان‌های بیماران و/یا اعضای خانواده، در کنار آن‌ها می‌مانم.	☐	☐	☐	☐	☐
۱۴. من می‌توانم "علائم هشداردهنده" نیازهای معنوی بیماران و/یا اعضای خانواده را حس کنم، مانند ابراز احساسات، ارزش‌ها یا تعارضات اخلاقی.	☐	☐	☐	☐	☐

۱۵. من با بیمار همکاری می‌کنم تا عناصر رضایت‌بخش که برای او ارزشمند است را پیدا کند.	☐	☐	☐	☐	☐
۱۶. من به بیماران و/یا اعضای خانواده کمک می‌کنم تا موقعیت‌های امیدهای واقعی را شناسایی کنند.	☐	☐	☐	☐	☐
۱۷. من به بیمار کمک می‌کنم تا مهم‌ترین رویدادهای زندگی خود را مرور کند.	☐	☐	☐	☐	☐
۱۸. . من به بیمار کمک می‌کنم تا تصمیمات یا خواسته‌های پایان زندگی را با پرسیدن سوالات باز یا خاص مشخص کند.	☐	☐	☐	☐	☐
۱۹. من بر ارتباط و هماهنگی بین تمام اعضای تیم و سطوح مختلف مراقبت تمرکز می‌کنم.	☐	☐	☐	☐	☐
۲۰. من بیماران و/یا اعضای خانواده را در طول روند بیماری به‌طور کافی از منابع موجود برای ادامهٔ مراقبت مطلع می‌سازم.	☐	☐	☐	☐	☐
