# Supplementary material for: Psychometric evaluation of the Palliative Nursing Care Quality Scale (PNCQS) among ICU and oncology nurses in Iran
Source: PLoS One. 2026 Nov 4;21(11):e0361384. doi: 10.1371/journal.pone.0361384 (PMC13734446; doi:10.1371/journal.pone.0361384)
Supplement: Supplementary file 2 [file pone.0361384.s002.docx]

| **S2 Table.** Impact Scores of the PNCQS Items | |
| --- | --- |
| Item | Impact score |
| 1. Perform a thorough assessment of pain, e.g. its location, frequency, intensity, and impact on the patient's quality of life. | 3.36 |
| 2. Provide the necessary means for the management of symptoms (anxiety, asthenia, confusion, dyspnea, pain, constipation, dry mouth, vomiting). | 3.44 |
| 3. Assess the effectiveness of care by asking the patient and/or family about their well-being or comfort. | 1.80 |
| 4. Encourage communication with family members and/or primary caregiver in a quiet and private place. | 2.28 |
| 5. Include family members and/or the primary caregiver in planning and executing care, depending on their disposition. | 3.60 |
| 6. Assess family and/or primary caregiver overload. | 2.04 |
| 7. Support family members and/or the primary caregiver in their work of caring for and accompanying the patient. | 2.34 |
| 8. Help the family and/or primary caregiver to express their feelings about loss and grief. | 1.61 |
| 9. Be aware of nonverbal messages from the patient and/or family, as well as words they prefer to avoid. | 1.51 |
| 10. Use active listening and silence for non-judgmental understanding of the feelings, thoughts and beliefs of the patient and/or family. | 2.28 |
| 11. Transmit understanding and warmth when the patient and/or family express feelings such as fear, anger or sadness. | 1.90 |
| 12. Help the patient and/or family to reflect on or clarify concerns about pending issues. | 1.66 |
| 13. Stay with the patient and/or family, especially during periods of greatest suffering. | 2.16 |
| 14. Detect "warning signs" of the spiritual needs of the patient and/or family such as expressions of emotion, values or ethical conflicts. | 1.66 |
| 15. Together with the patient, seek elements of satisfaction which have value for him/her. | 2.34 |
| 16. Help the patient and/or family identify situations of realistic hope. | 2.07 |
| 17. Help the patient when reviewing the most significant events of his/her life. | 1.74 |
| 18. Ask open and specific questions to support the patient in his/her decision making or final wishes. | 1.85 |
| 19. Ensure communication and coordination between all team members and the different levels of care. | 3.20 |
| 20. Adequately inform the patient/family throughout the process of the illness of the resources available for the continuity of care. | 2.28 |

S1 Table. Impact Scores of the PNCQS Items (Face Validity, n=20).
