# Supplementary material for: Psychometric evaluation of the Palliative Nursing Care Quality Scale (PNCQS) among ICU and oncology nurses in Iran
Source: PLoS One. 2026 Nov 4;21(11):e0361384. doi: 10.1371/journal.pone.0361384 (PMC13734446; doi:10.1371/journal.pone.0361384)
Supplement: Supplementary file 3 [file pone.0361384.s003.docx]

**S3 Table. Standardized Factor Loadings, Error Variances, and Latent Factor Correlations**

| **Factor** | **Item** | **Standardized Loading** | **Error Variance** |
| --- | --- | --- | --- |
| **F4** | q1 | 0.784 | 0.385 |
|  | q2 | 0.814 | 0.338 |
| **F1** | q3 | 0.590 | 0.652 |
|  | q4 | 0.672 | 0.548 |
|  | q5 | 0.691 | 0.522 |
|  | q6 | 0.649 | 0.578 |
|  | q7 | 0.694 | 0.518 |
|  | q8 | 0.645 | 0.583 |
| **F2** | q10 | 0.862 | 0.256 |
|  | q11 | 0.629 | 0.604 |
|  | q12 | 0.677 | 0.542 |
|  | q13 | 0.928 | 0.138 |
|  | q14 | 0.979 | 0.041 |
|  | q19 | 0.969 | 0.060 |
|  | q20 | 0.957 | 0.085 |
| **F3** | q9 | 0.451 | 0.797 |
|  | q15 | 0.973 | 0.054 |
|  | q16 | 0.945 | 0.107 |
|  | q17 | 0.699 | 0.511 |
|  | q18 | 0.950 | 0.097 |

**Factor Correlations:**

| **Factor Pair** | **Correlation** |
| --- | --- |
| F4 ↔ F1 | 0.375 |
| F4 ↔ F2 | 0.247 |
| F4 ↔ F3 | 0.218 |
| F1 ↔ F2 | 0.500 |
| F1 ↔ F3 | 0.432 |
| F2 ↔ F3 | 0.378 |

**Note:** All factor loadings and correlations are statistically significant (*p* < 0.001). Error variances were calculated as 1 – R².

**S2 Table.** Standardized Factor Loadings, Error Variances, and Latent Factor Correlations from the Confirmatory Factor Analysis (CFA) of the Persian PNCQS (n = 400).
