# Supplementary material for: Psychometric evaluation of the Palliative Nursing Care Quality Scale (PNCQS) among ICU and oncology nurses in Iran
Source: PLoS One. 2026 Nov 4;21(11):e0361384. doi: 10.1371/journal.pone.0361384 (PMC13734446; doi:10.1371/journal.pone.0361384)
Supplement: Supplementary file 4 [file pone.0361384.s005.docx]

**Response to the Editor and Reviewers**

We gratefully acknowledge the Editor and reviewers for their valuable comments. All points have been carefully addressed, and the corresponding revisions are marked using Track Changes in the revised manuscript. Below, we provide point‑by‑point responses to all comments..

Sincerely,

Corresponding author

| **Author’s responses** | **Comments of Editor** |
| --- | --- |
| We have carefully reviewed and followed the PLOS ONE style templates. The manuscript has been formatted accordingly, and all files are named as per the journal's guidelines. | 1. Please ensure that your manuscript meets PLOS ONE's style requirements, including those for file naming. The PLOS ONE style templates can be found at  https://journals.plos.org/plosone/s/file?id=ba62/PLOSOne_formatting_sample_title_authors_affiliations.pdf |
| The data availability statement has been revised. Requests for data should now be directed to the Tehran University of Medical Sciences Research Ethics Committee (ethics.research@tums.ac.ir), referencing ethics approval code IR.TUMS.FNM.REC.1403.170. This is a non-author institutional contact. | 2. In the online submission form you indicate that your data is not available for proprietary reasons and have provided a contact point for accessing this data. Please note that your current contact point is a co-author on this manuscript. According to our Data Policy, the contact point must not be an author on the manuscript and must be an institutional contact, ideally not an individual. Please revise your data statement to a non-author institutional point of contact, such as a data access or ethics committee, and send this to us via return email. Please also include contact information for the third party organization, and please include the full citation of where the data can be found. |
| The full ethics statement has been moved exclusively to the Methods section (subsection 2.1.3 Ethical Considerations) and completely removed from the Declarations section. | 3. Your ethics statement should only appear in the Methods section of your manuscript. If your ethics statement is written in any section besides the Methods, please move it to the Methods section and delete it from any other section. Please ensure that your ethics statement is included in your manuscript, as the ethics statement entered into the online submission form will not be published alongside your manuscript. |
| Captions for Supporting Information files (S1 Table and S2 Table) have been added at the end of the manuscript, and in-text citations have been updated accordingly. | 4. Please include captions for your Supporting Information files at the end of your manuscript, and update any in-text citations to match accordingly. Please see our Supporting Information guidelines for more information: http://journals.plos.org/plosone/s/supporting-information. |
| We appreciate the editor's acknowledgment. | 5. Thank you for providing your underlying data as Supporting Information. |
| After carefully reviewing the reviewer comments, we found that no specific previously published works were recommended for citation. | 6. If the reviewer comments include a recommendation to cite specific previously published works, please review and evaluate these publications to determine whether they are relevant and should be cited. There is no requirement to cite these works unless the editor has indicated otherwise. |
| **Author’s responses** | **Additional Editor Comments:** |
| **Introduction** | |
| Thank you for this valuable suggestion. A dedicated paragraph has been added to the Introduction explicitly justifying the expected multidimensional structure. This paragraph explains how Iran's collectivist culture, strong family involvement, and spiritual-religious values may lead nurses to perceive palliative care quality through distinct yet interconnected dimensions, in contrast to the original unidimensional structure developed in a Western context. These changes are highlighted in red in the revised manuscript. | 1. The introduction is generally well‑structured, but the conceptual justification for the four‑factor structure emerges only in the Discussion. The manuscript would benefit from stronger theoretical grounding earlier, especially given the shift from the original unidimensional structure. Add a paragraph in the Introduction explaining why multidimensionality may emerge in collectivist, family‑centric, and spiritually oriented contexts. |
| **Methods** | |
| We have clarified the sampling procedure in the Methods section (subsection 2.1.1) . The multi‑stage cluster sampling is fully described, and we explicitly acknowledge that the use of convenience sampling at the final stage may have introduced selection bias, potentially limiting generalizability. Since demographic data on non‑participants were not systematically collected, a formal comparison was not feasible. This limitation is transparently discussed in the Discussion section (Limitations and future directions) with recommendations for future studies. | 2. The authors use multi‑stage cluster sampling but then rely on convenience sampling within wards, which introduces bias. It is recommended that the authors clarify how convenience sampling may affect generalizability and whether demographic comparisons were made between included and excluded nurses. |
| **Result** | |
| We thank the editor for this comment. Upon re‑checking, the lowest loading was 0.42 (not 0.38). The value 0.38 was a typographical error, which has been corrected to 0.42 and 0.48 in Table 3. A clear rationale for retaining these two lower‑loading items has been provided in the Results section (Step 2 – EFA) , emphasizing their theoretical relevance to the construct in the Iranian cultural and spiritual context, their contribution to content validity, and their minimal negative impact on overall reliability and model fit. | 3. The EFA results show several items with borderline factor loadings (e.g., 0.38, 0.40). The authors retained all items, but justification is limited. Provide a rationale for retaining items with low loadings, including theoretical relevance or cross‑cultural considerations. |
| We have expanded the justification in Section 3.5 (Convergent and discriminant validity, construct reliability) . We now report that despite AVE = 0.42 for the Family‑Centered Care factor, composite reliability (CR) remained acceptable (0.81), and all standardized factor loadings for this factor were significant and ranged from 0.52 to 0.78 (p < 0.001), confirming that the low AVE is not due to weak or problematic items. | 4. The Family Centered Care factor has an AVE of 0.42, below the recommended 0.50 threshold. I will encourage the authors to expand the justification and consider reporting item level loadings for this factor to demonstrate that low AVE is not due to problematic items. |
| The inter‑item correlation (r = 0.77) and Spearman‑Brown coefficient (0.77) have been added in Section 3.6 (Reliability) . We have also clarified the content coverage and theoretical importance of the two items, explaining why this brief subscale remains meaningful and representative of the construct. | 5. The Physical Symptom Management subscale contains only two items. Cronbach’s alpha for two item scales is known to be unstable. The authors should consider reporting inter item correlation (r) and Spearman Brown coefficient. |
| A new Supplementary Table S2 containing standardized factor loadings, error variances, and latent factor correlations has been added and cited in the Results section (Step 3 – CFA). | 6. The CFA results are strong, but the authors do not provide standardized factor loadings table, error variances, and correlations between latent factors. These are critical and should be included in the CFA results. Perhaps, the authors can include a supplementary table with full CFA parameter estimates. |
| The ethics statement in subsection 2.1.3 (Ethical Considerations) has been expanded to explicitly include: (1) participation was voluntary and anonymous; (2) no financial or non‑financial incentives were provided; and (3) potential coercion in hierarchical hospital settings was mitigated through independent researcher administration and clear assurance of no negative professional consequences. | 7. The ethics statement is thorough, but the Methods section should explicitly state whether participation was anonymous or confidential, whether any incentives were provided, and how potential coercion was mitigated in hierarchical hospital settings. |
| The entire manuscript has been thoroughly checked and edited for language and grammar by a professional English editor. We believe the revised text now meets the journal's standards. | 8. As a minor comment, i will encourage the authors to have the manuscript checked for language usage as there are some grammatical errors. |
| **Response to Comments of Reviewer 1** | **Comments of Reviewer 1** |
| We sincerely thank the reviewer for the positive and supportive feedback. We are truly encouraged by the acknowledgment of our study's importance, methodological rigor, and large sample size. We greatly appreciate the reviewer's agreement with the manuscript. | 1. The study is essential for palliative care patient. The importance of the study is were used in two of factor analysis which is the confirmation and exploration. The advantage of the study was participates 620 nurses. Therefore I am agree that the manucript |
| **Response to Comments of Reviewer 2** | **Comments of Reviewer 2** |
| **Abstract** | |
| The timeline has been unified to "August 2025 to April 2026" in both the Abstract and the Methods section. This change is highlighted in red. | Please address the following queries  1. Inconsistency in Study Timeline  Relook into the timeline as it is different in abstract and methods, and make it uniform as either 2025 or August 2025 to April 2026 |
| **Introduction** | |
| The Introduction has been revised to sharpen the literature gap. We now emphasize: (1) the lack of a Persian‑validated PNCQS; (2) the limitations of existing scales, which are primarily patient‑ or family‑focused and restricted to specific populations; and (3) the absence of nurse‑driven quality assessment tools in Iran. | 1. Strengthen the Literature Gap Sharpen the literature by emphasizing mainly on the Persian version of the tool availability, existing scales' limitations to assess the outcome of nursing care quality, and the lack of nurse-driven quality assessment tools. |
| **Methods** | |
| We performed the initial CFA (n=220) on the original model to test its applicability in the Iranian context. After poor fit, EFA was conducted on the same sample to identify a culturally relevant structure. We acknowledge this as a limitation in the Discussion. However, the final CFA of the four‑factor model was performed on an entirely independent sample (n=400), ensuring robust cross‑validation. This approach is methodologically justifiable and practically sound. Full justification is provided in section 2.1.1 (Sample size determination). | 1. Same Sample Used for EFA and Initial CFA with 220 nurses. Please justify the selection of the same sample for the initial CFA of the original model and for the EFA of the revised structure. Or acknowledge the same as a limitation in the study. |
| **Results** | |
| We have addressed this comment in Section 3.6 (Reliability) by reporting the inter‑item correlation (r = 0.77) and Spearman‑Brown coefficient (0.77). We also clarified that although this subscale contains only two items, they assess core aspects of physical symptom management (pain control and overall symptom relief) and demonstrate acceptable reliability. This is consistent with previous studies showing that well‑designed two‑item subscales can demonstrate adequate reliability when supplemented with Spearman‑Brown correction. | 1. Weak Physical Symptom Management Factor The factor contains only two items. Please clarify the stability of the factor, the coverage of the content, and the representation of the construct for the 2 items above. |
| Supplementary Table S1, presenting impact scores for all 20 items, has been added to improve the visibility and transparency of the face validity data. This table is cited in the Results section (3.2 Face Validity). | 2. Add Table for Face Validity Add a supplementary table with the item and impact scores to improve the visibility and transparency of the data |
| The loading of 0.38 was a typographical error; it has been corrected to 0.42 and 0.48 in Table 3. All loadings are now ≥ 0.42. A clear rationale for retaining these two lower‑loading items (theoretical relevance and cross‑cultural considerations) has been provided in the Results section (Step 2 – EFA). | 3. Factor Loadings of 0.38. Inconsistency between the table 3 report and the methods section needs to be addressed. Either remove from one section or justify why it is removed. |
| **Discussion** | |
| The Discussion has been strengthened with a detailed explanation of the suboptimal AVE for Family‑Centered Care. We now discuss how cultural complexity and heterogeneity in family‑centered care practices within the Iranian context—including shared decision‑making, emotional containment, advocacy, and indirect spiritual support—may generate greater response variability among nurses. This heterogeneity reflects the construct's rich and context‑dependent manifestation rather than a methodological flaw. Future refinement of this subscale is encouraged. | 1. Family-Centered Care AVE Below Standard There is a brief explanation for this aspect as AVE = 0.42 (<0.50). clarify this by strengthening the discussion with details on cultural complexity, the heterogeneity of family-centered care, and the future need for any restructuring. |
| **Conclusion** | |
| Due to ethical restrictions and institutional policies protecting participant confidentiality, the dataset cannot be deposited in a public repository. However, the data are available upon reasonable request from the Tehran University of Medical Sciences Research Ethics Committee (ethics.research@tums.ac.ir), referencing ethics approval code IR.TUMS.FNM.REC.1403.170. This statement has been included in the Data Availability section. | 5. Data sharing should be shown in repositories |

1. Is the manuscript technically sound, and do the data support the conclusions?

The manuscript must describe a technically sound piece of scientific research with data that supports the conclusions. Experiments must have been conducted rigorously, with appropriate controls, replication, and sample sizes. The conclusions must be drawn appropriately based on the data presented.

Reviewer #1: Yes

Reviewer #2: Yes

2. Has the statistical analysis been performed appropriately and rigorously?

Reviewer #1: Yes

Reviewer #2: Yes

3. Have the authors made all data underlying the findings in their manuscript fully available?

The [PLOS Data policy](http://track.editorialmanager.com/CL0/http:%2F%2Fwww.plosone.org%2Fstatic%2Fpolicies.action%23sharing/1/010f019ec9e67d15-f570bf00-4547-4113-b140-6b4fc7c59ca0-000000/PgjvuHypA_j7hVaVuTicUXzP7HpNgjDFKVq_GIDI72U=258) requires authors to make all data underlying the findings described in their manuscript fully available without restriction, with rare exception (please refer to the Data Availability Statement in the manuscript PDF file). The data should be provided as part of the manuscript or its supporting information, or deposited to a public repository. For example, in addition to summary statistics, the data points behind means, medians and variance measures should be available. If there are restrictions on publicly sharing data—e.g. participant privacy or use of data from a third party—those must be specified.

Reviewer #1: Yes

Reviewer #2: No

4. Is the manuscript presented in an intelligible fashion and written in standard English?

PLOS ONE does not copyedit accepted manuscripts, so the language in submitted articles must be clear, correct, and unambiguous. Any typographical or grammatical errors should be corrected at revision, so please note any specific errors here.

Reviewer #1: Yes

Reviewer #2: Yes
