# Supplementary material for: Psychometric evaluation of the Palliative Nursing Care Quality Scale (PNCQS) among ICU and oncology nurses in Iran
Source: PLoS One. 2026 Nov 4;21(11):e0361384. doi: 10.1371/journal.pone.0361384 (PMC13734446; doi:10.1371/journal.pone.0361384)
Supplement: Supplementary file 5 [file pone.0361384.s006.docx]

**Response to the Editor and Reviewers**

We gratefully acknowledge the Editor and reviewers for their valuable comments and constructive feedback. All comments have been carefully addressed, and the corresponding revisions have been incorporated into the revised manuscript. All changes are highlighted using Track Changes. Below, we provide a point-by-point response to each comment.

Sincerely,

Corresponding Author

| **Author’s responses** | **Comments of Editor** |
| --- | --- |
| Thank you for this comment. We carefully reviewed all reviewer comments and confirmed that no specific publications were recommended for citation. Nevertheless, we reviewed the recent literature and updated the reference list where appropriate. | If the reviewer comments include a recommendation to cite specific previously published works, please review and evaluate these publications to determine whether they are relevant and should be cited. There is no requirement to cite these works unless the editor has indicated otherwise. |
| Thank you for this valuable comment. We carefully reviewed the entire reference list and confirmed that all references are complete, accurate, and up to date. We also verified that none of the cited articles have been retracted. Minor formatting corrections were made to ensure consistency with the journal style. | Please review your reference list to ensure that it is complete and correct. If you have cited papers that have been retracted, please include the rationale for doing so in the manuscript text, or remove these references and replace them with relevant current references. Any changes to the reference list should be mentioned in the rebuttal letter that accompanies your revised manuscript. If you need to cite a retracted article, indicate the article’s retracted status in the References list and also include a citation and full reference for the retraction notice. |
| **Response to Comments of Reviewer 2** | **Comments of Reviewer 2** |
| 1- Thank you for the suggestion. The title has been revised accordingly. 2- Thank you. The EFA was repeated using Direct Oblimin rotation, and the manuscript has been revised accordingly. 3- Thank you. Parallel analysis was performed in R and confirmed the four-factor solution. The results and figure have been added to the manuscript. 4- Thank you. As recommended, the absence of multigroup CFA has been acknowledged in the Limitations section. 5- Thank you. This point has been clarified in the Methods section by stating that no validated Persian gold-standard instrument was available for criterion validity assessment. 6- Thank you. We have added a justification for using maximum likelihood estimation with bootstrapping in the Preliminary Data Checks subsection. 7- Thank you. The terminology has been revised throughout the manuscript to ensure consistent capitalization. | Reviewer #2: Suggestions for improvement   1- Title it is written as Iranian nurses, consider writing ICU and oncology nurses from Iran 2- EFA should use oblique rotation instead of Varimax 3- Perform Parallel Analysis to support the four-factor solution. 4- Include multigroup CFA or acknowledge this as a limitation. 5- Explain why criterion validity could not be evaluated because no Persian gold standard exists. 6- Please justify ML estimation as the items are ordinal 7- Please maintain consistent capitalization of family-centered care in the manuscript |
