# Supplementary material for: Psychometric evaluation of the Palliative Nursing Care Quality Scale (PNCQS) among ICU and oncology nurses in Iran
Source: PLoS One. 2026 Nov 4;21(11):e0361384. doi: 10.1371/journal.pone.0361384 (PMC13734446; doi:10.1371/journal.pone.0361384)
Supplement: Peer review history

# Peer review: Decision Letter 0

**DOI:** 10.1371/journal.pone.0361384.r001

**Authors:** JONATHAN BAYUO

15 Jun 2026

Dear Dr. Shahmari,

Thank you for submitting your manuscript to PLOS ONE. After careful consideration, we feel that it has merit but does not fully meet PLOS ONE’s publication criteria as it currently stands. Therefore, we invite you to submit a revised version of the manuscript that addresses the points raised during the review process.

Please submit your revised manuscript by Aug 14 2026 11:59PM. If you will need more time than this to complete your revisions, please reply to this message or contact the journal office at plosone@plos.org. When you're ready to submit your revision, log on to https://www.editorialmanager.com/pone/ and select the 'Submissions Needing Revision' folder to locate your manuscript file.

- A letter that responds to each point raised by the academic editor and reviewer(s). You should upload this letter as a separate file labeled 'Response to Reviewers'.
- A marked-up copy of your manuscript that highlights changes made to the original version. You should upload this as a separate file labeled 'Revised Manuscript with Track Changes'.
- An unmarked version of your revised paper without tracked changes. You should upload this as a separate file labeled 'Manuscript'.

If you would like to make changes to your financial disclosure, please include your updated statement in your cover letter. Guidelines for resubmitting your figure files are available below the reviewer comments at the end of this letter.

If applicable, we recommend that you deposit your laboratory protocols in protocols.io to enhance the reproducibility of your results. Protocols.io assigns your protocol its own identifier (DOI) so that it can be cited independently in the future. For instructions see: https://journals.plos.org/plosone/s/submission-guidelines#loc-laboratory-protocols. Additionally, PLOS ONE offers an option for publishing peer-reviewed Lab Protocol articles, which describe protocols hosted on protocols.io. Read more information on sharing protocols at https://plos.org/protocols?utm_medium=editorial-email&utm_source=authorletters&utm_campaign=protocols.

As the corresponding author, your ORCID iD is verified in the submission system and will appear in the published article. PLOS supports the use of ORCID, and we encourage all coauthors to register for an ORCID iD and use it as well. Please encourage your coauthors to verify their ORCID iD within the submission system before final acceptance, as unverified ORCID iDs will not appear in the published article. *Only*  the individual author can complete the verification step; PLOS staff *cannot*  verify ORCID iDs on behalf of authors.

We look forward to receiving your revised manuscript.

Kind regards,

JONATHAN BAYUO, PhD

Academic Editor

PLOS One

**Journal Requirements:**

When submitting your revision, we need you to address these additional requirements.

1. Please ensure that your manuscript meets PLOS ONE's style requirements, including those for file naming. The PLOS ONE style templates can be found at

https://journals.plos.org/plosone/s/file?id=wjVg/PLOSOne_formatting_sample_main_body.pdf and

https://journals.plos.org/plosone/s/file?id=ba62/PLOSOne_formatting_sample_title_authors_affiliations.pdf

2. In the online submission form you indicate that your data is not available for proprietary reasons and have provided a contact point for accessing this data. Please note that your current contact point is a co-author on this manuscript. According to our Data Policy, the contact point must not be an author on the manuscript and must be an institutional contact, ideally not an individual. Please revise your data statement to a non-author institutional point of contact, such as a data access or ethics committee, and send this to us via return email. Please also include contact information for the third party organization, and please include the full citation of where the data can be found.

3. Your ethics statement should only appear in the Methods section of your manuscript. If your ethics statement is written in any section besides the Methods, please move it to the Methods section and delete it from any other section. Please ensure that your ethics statement is included in your manuscript, as the ethics statement entered into the online submission form will not be published alongside your manuscript.

4. Please include captions for your Supporting Information files at the end of your manuscript, and update any in-text citations to match accordingly. Please see our Supporting Information guidelines for more information: http://journals.plos.org/plosone/s/supporting-information.

5. Thank you for providing your underlying data as Supporting Information.

We note that the data set contains text or data that is not in English. Please note that PLOS is an English-language publisher, so we require data sets to be provided in English as well. Please upload an English-language version of your data set.

This will also allow us to determine if your data follows PLOS standards per our Data Availability policy here: https://journals.plos.org/plosone/s/data-availability

6. If the reviewer comments include a recommendation to cite specific previously published works, please review and evaluate these publications to determine whether they are relevant and should be cited. There is no requirement to cite these works unless the editor has indicated otherwise.

**Additional Editor Comments:**

The authors present a methodological study evaluating the psychometric properties of the Persian version of the Palliative Nursing Care Quality Scale (PNCQS). The study includes translation following WHO guidelines, face/content validity assessment, exploratory and confirmatory factor analyses, and reliability testing among 620 Iranian nurses. The authors report a four‑factor structure—Family‑Centered Care, Empathic and Spiritual Care, Hope and Life Review, and Physical Symptom Management—explaining 51.41% of variance, with strong reliability and acceptable validity indices. The manuscript addresses an important gap in palliative care measurement in Iran and contributes a culturally adapted tool for clinical and research use. Kindly consider the following comments in addition to those raised by the reviewers:

1. The introduction is generally well‑structured, but the conceptual justification for the four‑factor structure emerges only in the Discussion. The manuscript would benefit from stronger theoretical grounding earlier, especially given the shift from the original unidimensional structure. Add a paragraph in the Introduction explaining why multidimensionality may emerge in collectivist, family‑centric, and spiritually oriented contexts.

2. The authors use multi‑stage cluster sampling but then rely on convenience sampling within wards, which introduces bias. It is recommended that the authors clarify how convenience sampling may affect generalizability and whether demographic comparisons were made between included and excluded nurses.

3. The EFA results show several items with borderline factor loadings (e.g., 0.38, 0.40). The authors retained all items, but justification is limited. Provide a rationale for retaining items with low loadings, including theoretical relevance or cross‑cultural considerations.

4. The Family‑Centered Care factor has an AVE of 0.42, below the recommended 0.50 threshold. I will encourage the authors to expand the justification and consider reporting item‑level loadings for this factor to demonstrate that low AVE is not due to problematic items.

5. The Physical Symptom Management subscale contains only two items. Cronbach’s alpha for two‑item scales is known to be unstable. The authors should consider reporting inter‑item correlation (r) and Spearman‑Brown coefficient.

6. The CFA results are strong, but the authors do not provide standardized factor loadings table, error variances, and correlations between latent factors. These are critical and should be included in the CFA results. Perhaps, the authors can include a supplementary table with full CFA parameter estimates.

7. The ethics statement is thorough, but the Methods section should explicitly state whether participation was anonymous or confidential, whether any incentives were provided, and how potential coercion was mitigated in hierarchical hospital settings.

As a minor comment, i will encourage the authors to have the manuscript checked for language usage as there are some grammatical errors.

[Note: HTML markup is below. Please do not edit.]

Reviewers' comments:

Reviewer's Responses to Questions

**Comments to the Author**

1. Is the manuscript technically sound, and do the data support the conclusions?

Reviewer #1: Yes

Reviewer #2: Yes

**********

2. Has the statistical analysis been performed appropriately and rigorously?

Reviewer #1: Yes

Reviewer #2: Yes

**********

3. Have the authors made all data underlying the findings in their manuscript fully available?

The PLOS Data policy

Reviewer #1: Yes

Reviewer #2: No

**********

4. Is the manuscript presented in an intelligible fashion and written in standard English?

Reviewer #1: Yes

Reviewer #2: Yes

**********

Reviewer #1: The study is essential for palliative care patient. The importance of the study is were used in two of factor analysis which is the confirmation and exploration. The advantage of the study was participates 620 nurses. Therefore I am agree that the manucript

Reviewer #2: Please address the following queries

1. Inconsistency in Study Timeline

Relook into the timeline as it is different in abstract and methods, and make it uniform as either 2025 or August 2025 to April 2026

2. Methods

Same Sample Used for EFA and Initial CFA with 220 nurses. Please justify the selection of the same sample for the initial CFA of the original model and for the EFA of the revised structure. Or acknowledge the same as a limitation in the study.

3. Weak Physical Symptom Management Factor

The factor contains only two items. Please clarify the stability of the factor, the coverage of the content, and the representation of the construct for the 2 items above.

4. Family-Centered Care AVE Below Standard

There is a brief explanation for this aspect as AVE = 0.42 (<0.50). clarify this by strengthening the discussion with details on cultural complexity, the heterogeneity of family-centered care, and the future need for any restructuring.

5. Data sharing should be shown in repositories

6. Strengthen the Literature Gap

Sharpen the literature by emphasizing mainly on the Persian version of the tool availability, existing scales' limitations to assess the outcome of nursing care quality, and the lack of nurse-driven quality assessment tools.

7. Add Table for Face Validity

Add a supplementary table with the item and impact scores to improve the visibility and transparency of the data

8. Factor Loadings of 0.38. Inconsistency between the table 3 report and the methods section needs to be addressed. Either remove from one section or justify why it is removed.

**********

what does this mean?). If published, this will include your full peer review and any attached files.

If you choose “no”, your identity will remain anonymous but your review may still be made public.

**Do you want your identity to be public for this peer review?**  For information about this choice, including consent withdrawal, please see our Privacy Policy

Reviewer #1: **Yes:**  Gelegjamt Delgersuren

Reviewer #2: No

**********

[NOTE: If reviewer comments were submitted as an attachment file, they will be attached to this email and accessible via the submission site. Please log into your account, locate the manuscript record, and check for the action link "View Attachments". If this link does not appear, there are no attachment files.]

To ensure your figures meet our technical requirements, please review our figure guidelines: https://journals.plos.org/plosone/s/figures

You may also use PLOS’s free figure tool, NAAS, to help you prepare publication quality figures: https://journals.plos.org/plosone/s/figures#loc-tools-for-figure-preparation.

NAAS will assess whether your figures meet our technical requirements by comparing each figure against our figure specifications.

# Author comment: Author response to Decision Letter 1

**DOI:** 10.1371/journal.pone.0361384.r002

26 Jun 2026

Response to the Editor and Reviewers

We gratefully acknowledge the Editor and reviewers for their valuable comments. All points have been carefully addressed, and the corresponding revisions are marked using Track Changes in the revised manuscript. Below, we provide point‑by‑point responses to all comments..

Sincerely,

Corresponding author

Author’s responses Comments of Editor

We have carefully reviewed and followed the PLOS ONE style templates. The manuscript has been formatted accordingly, and all files are named as per the journal's guidelines. 1. Please ensure that your manuscript meets PLOS ONE's style requirements, including those for file naming. The PLOS ONE style templates can be found at

https://journals.plos.org/plosone/s/file?id=ba62/PLOSOne_formatting_sample_title_authors_affiliations.pdf

The data availability statement has been revised. Requests for data should now be directed to the Tehran University of Medical Sciences Research Ethics Committee (ethics.research@tums.ac.ir), referencing ethics approval code IR.TUMS.FNM.REC.1403.170. This is a non-author institutional contact. 2. In the online submission form you indicate that your data is not available for proprietary reasons and have provided a contact point for accessing this data. Please note that your current contact point is a co-author on this manuscript. According to our Data Policy, the contact point must not be an author on the manuscript and must be an institutional contact, ideally not an individual. Please revise your data statement to a non-author institutional point of contact, such as a data access or ethics committee, and send this to us via return email. Please also include contact information for the third party organization, and please include the full citation of where the data can be found.

The full ethics statement has been moved exclusively to the Methods section (subsection 2.1.3 Ethical Considerations) and completely removed from the Declarations section. 3. Your ethics statement should only appear in the Methods section of your manuscript. If your ethics statement is written in any section besides the Methods, please move it to the Methods section and delete it from any other section. Please ensure that your ethics statement is included in your manuscript, as the ethics statement entered into the online submission form will not be published alongside your manuscript.

Captions for Supporting Information files (S1 Table and S2 Table) have been added at the end of the manuscript, and in-text citations have been updated accordingly. 4. Please include captions for your Supporting Information files at the end of your manuscript, and update any in-text citations to match accordingly. Please see our Supporting Information guidelines for more information: http://journals.plos.org/plosone/s/supporting-information.

We appreciate the editor's acknowledgment. 5. Thank you for providing your underlying data as Supporting Information.

After carefully reviewing the reviewer comments, we found that no specific previously published works were recommended for citation. 6. If the reviewer comments include a recommendation to cite specific previously published works, please review and evaluate these publications to determine whether they are relevant and should be cited. There is no requirement to cite these works unless the editor has indicated otherwise.

Author’s responses Additional Editor Comments:

Introduction

Thank you for this valuable suggestion. A dedicated paragraph has been added to the Introduction explicitly justifying the expected multidimensional structure. This paragraph explains how Iran's collectivist culture, strong family involvement, and spiritual-religious values may lead nurses to perceive palliative care quality through distinct yet interconnected dimensions, in contrast to the original unidimensional structure developed in a Western context. These changes are highlighted in red in the revised manuscript. 1. The introduction is generally well‑structured, but the conceptual justification for the four‑factor structure emerges only in the Discussion. The manuscript would benefit from stronger theoretical grounding earlier, especially given the shift from the original unidimensional structure. Add a paragraph in the Introduction explaining why multidimensionality may emerge in collectivist, family‑centric, and spiritually oriented contexts.

Methods

We have clarified the sampling procedure in the Methods section (subsection 2.1.1) . The multi‑stage cluster sampling is fully described, and we explicitly acknowledge that the use of convenience sampling at the final stage may have introduced selection bias, potentially limiting generalizability. Since demographic data on non‑participants were not systematically collected, a formal comparison was not feasible. This limitation is transparently discussed in the Discussion section (Limitations and future directions) with recommendations for future studies. 2. The authors use multi‑stage cluster sampling but then rely on convenience sampling within wards, which introduces bias. It is recommended that the authors clarify how convenience sampling may affect generalizability and whether demographic comparisons were made between included and excluded nurses.

Result

We thank the editor for this comment. Upon re‑checking, the lowest loading was 0.42 (not 0.38). The value 0.38 was a typographical error, which has been corrected to 0.42 and 0.48 in Table 3. A clear rationale for retaining these two lower‑loading items has been provided in the Results section (Step 2 – EFA) , emphasizing their theoretical relevance to the construct in the Iranian cultural and spiritual context, their contribution to content validity, and their minimal negative impact on overall reliability and model fit. 3. The EFA results show several items with borderline factor loadings (e.g., 0.38, 0.40). The authors retained all items, but justification is limited. Provide a rationale for retaining items with low loadings, including theoretical relevance or cross cultural considerations.

We have expanded the justification in Section 3.5 (Convergent and discriminant validity, construct reliability) . We now report that despite AVE = 0.42 for the Family‑Centered Care factor, composite reliability (CR) remained acceptable (0.81), and all standardized factor loadings for this factor were significant and ranged from 0.52 to 0.78 (p < 0.001), confirming that the low AVE is not due to weak or problematic items. 4. The Family Centered Care factor has an AVE of 0.42, below the recommended 0.50 threshold. I will encourage the authors to expand the justification and consider reporting item level loadings for this factor to demonstrate that low AVE is not due to problematic items.

The inter‑item correlation (r = 0.77) and Spearman‑Brown coefficient (0.77) have been added in Section 3.6 (Reliability) . We have also clarified the content coverage and theoretical importance of the two items, explaining why this brief subscale remains meaningful and representative of the construct. 5. The Physical Symptom Management subscale contains only two items. Cronbach’s alpha for two item scales is known to be unstable. The authors should consider reporting inter item correlation (r) and Spearman Brown coefficient.

A new Supplementary Table S2 containing standardized factor loadings, error variances, and latent factor correlations has been added and cited in the Results section (Step 3 – CFA). 6. The CFA results are strong, but the authors do not provide standardized factor loadings table, error variances, and correlations between latent factors. These are critical and should be included in the CFA results. Perhaps, the authors can include a supplementary table with full CFA parameter estimates.

The ethics statement in subsection 2.1.3 (Ethical Considerations) has been expanded to explicitly include: (1) participation was voluntary and anonymous; (2) no financial or non‑financial incentives were provided; and (3) potential coercion in hierarchical hospital settings was mitigated through independent researcher administration and clear assurance of no negative professional consequences. 7. The ethics statement is thorough, but the Methods section should explicitly state whether participation was anonymous or confidential, whether any incentives were provided, and how potential coercion was mitigated in hierarchical hospital settings.

The entire manuscript has been thoroughly checked and edited for language and grammar by a professional English editor. We believe the revised text now meets the journal's standards. 8. As a minor comment, i will encourage the authors to have the manuscript checked for language usage as there are some grammatical errors.

Response to Comments of Reviewer 1 Comments of Reviewer 1

We sincerely thank the reviewer for the positive and supportive feedback. We are truly encouraged by the acknowledgment of our study's importance, methodological rigor, and large sample size. We greatly appreciate the reviewer's agreement with the manuscript. 1. The study is essential for palliative care patient. The importance of the study is were used in two of factor analysis which is the confirmation and exploration. The advantage of the study was participates 620 nurses. Therefore I am agree that the manucript

Response to Comments of Reviewer 2 Comments of Reviewer 2

Abstract

The timeline has been unified to "August 2025 to April 2026" in both the Abstract and the Methods section. This change is highlighted in red. Please address the following queries

1. Inconsistency in Study Timeline

Relook into the timeline as it is different in abstract and methods, and make it uniform as either 2025 or August 2025 to April 2026

Introduction

The Introduction has been revised to sharpen the literature gap. We now emphasize: (1) the lack of a Persian‑validated PNCQS; (2) the limitations of existing scales, which are primarily patient‑ or family‑focused and restricted to specific populations; and (3) the absence of nurse‑driven quality assessment tools in Iran. 1. Strengthen the Literature Gap

Sharpen the literature by emphasizing mainly on the Persian version of the tool availability, existing scales' limitations to assess the outcome of nursing care quality, and the lack of nurse-driven quality assessment tools.

Methods

We performed the initial CFA (n=220) on the original model to test its applicability in the Iranian context. After poor fit, EFA was conducted on the same sample to identify a culturally relevant structure. We acknowledge this as a limitation in the Discussion. However, the final CFA of the four‑factor model was performed on an entirely independent sample (n=400), ensuring robust cross‑validation. This approach is methodologically justifiable and practically sound. Full justification is provided in section 2.1.1 (Sample size determination). 1. Same Sample Used for EFA and Initial CFA with 220 nurses. Please justify the selection of the same sample for the initial CFA of the original model and for the EFA of the revised structure. Or acknowledge the same as a limitation in the study.

Results

We have addressed this comment in Section 3.6 (Reliability) by reporting the inter‑item correlation (r = 0.77) and Spearman‑Brown coefficient (0.77). We also clarified that although this subscale contains only two items, they assess core aspects of physical symptom management (pain control and overall symptom relief) and demonstrate acceptable reliability. This is consistent with previous studies showing that well‑designed two‑item subscales can demonstrate adequate reliability when supplemented with Spearman‑Brown correction. 1. Weak Physical Symptom Management Factor

The factor contains only two items. Please clarify the stability of the factor, the coverage of the content, and the representation of the construct for the 2 items above.

Supplementary Table S1, presenting impact scores for all 20 items, has been added to improve the visibility and transparency of the face validity data. This table is cited in the Results section (3.2 Face Validity). 2. Add Table for Face Validity

Add a supplementary table with the item and impact scores to improve the visibility and transparency of the data

The loading of 0.38 was a typographical error; it has been corrected to 0.42 and 0.48 in Table 3. All loadings are now ≥ 0.42. A clear rationale for retaining these two lower‑loading items (theoretical relevance and cross‑cultural considerations) has been provided in the Results section (Step 2 – EFA). 3. Factor Loadings of 0.38. Inconsistency between the table 3 report and the methods section needs to be addressed. Either remove from one section or justify why it is removed.

Discussion

The Discussion has been strengthened with a detailed explanation of the suboptimal AVE for Family‑Centered Care. We now discuss how cultural complexity and heterogeneity in family‑centered care practices within the Iranian context—including shared decision‑making, emotional containment, advocacy, and indirect spiritual support—may generate greater response variability among nurses. This heterogeneity reflects the construct's rich and context‑dependent manifestation rather than a methodological flaw. Future refinement of this subscale is encouraged. 1. Family-Centered Care AVE Below Standard

There is a brief explanation for this aspect as AVE = 0.42 (<0.50). clarify this by strengthening the discussion with details on cultural complexity, the heterogeneity of family-centered care, and the future need for any restructuring.

Conclusion

Due to ethical restrictions and institutional policies protecting participant confidentiality, the dataset cannot be deposited in a public repository. However, the data are available upon reasonable request from the Tehran University of Medical Sciences Research Ethics Committee (ethics.research@tums.ac.ir), referencing ethics approval code IR.TUMS.FNM.REC.1403.170. This statement has been included in the Data Availability section. 5. Data sharing should be shown in repositories

1. Is the manuscript technically sound, and do the data support the conclusions?

The manuscript must describe a technically sound piece of scientific research with data that supports the conclusions. Experiments must have been conducted rigorously, with appropriate controls, replication, and sample sizes. The conclusions must be drawn appropriately based on the data presented.

Reviewer #1: Yes

Reviewer #2: Yes

________________________________________

2. Has the statistical analysis been performed appropriately and rigorously?

Reviewer #1: Yes

Reviewer #2: Yes

________________________________________

3. Have the authors made all data underlying the findings in their manuscript fully available?

The PLOS Data policy requires authors to make all data underlying the findings described in their manuscript fully available without restriction, with rare exception (please refer to the Data Availability Statement in the manuscript PDF file). The data should be provided as part of the manuscript or its supporting information, or deposited to a public repository. For example, in addition to summary statistics, the data points behind means, medians and variance measures should be available. If there are restrictions on publicly sharing data—e.g. participant privacy or use of data from a third party—those must be specified.

Reviewer #1: Yes

Reviewer #2: No

________________________________________

4. Is the manuscript presented in an intelligible fashion and written in standard English?

PLOS ONE does not copyedit accepted manuscripts, so the language in submitted articles must be clear, correct, and unambiguous. Any typographical or grammatical errors should be corrected at revision, so please note any specific errors here.

Reviewer #1: Yes

Reviewer #2: Yes

Attachment Submitted filename: response to reviewers.docx

# Peer review: Decision Letter 1

**DOI:** 10.1371/journal.pone.0361384.r003

**Authors:** JONATHAN BAYUO

15 Jul 2026

Dear Dr. Shahmari,

Thank you for submitting your manuscript to PLOS ONE. After careful consideration, we feel that it has merit but does not fully meet PLOS ONE’s publication criteria as it currently stands. Therefore, we invite you to submit a revised version of the manuscript that addresses the points raised during the review process.

Please submit your revised manuscript by Aug 14 2026 11:59PM. If you will need more time than this to complete your revisions, please reply to this message or contact the journal office at plosone@plos.org. When you're ready to submit your revision, log on to https://www.editorialmanager.com/pone/ and select the 'Submissions Needing Revision' folder to locate your manuscript file.

- A letter that responds to each point raised by the academic editor and reviewer(s). You should upload this letter as a separate file labeled 'Response to Reviewers'.
- A marked-up copy of your manuscript that highlights changes made to the original version. You should upload this as a separate file labeled 'Revised Manuscript with Track Changes'.
- An unmarked version of your revised paper without tracked changes. You should upload this as a separate file labeled 'Manuscript'.

If you would like to make changes to your financial disclosure, please include your updated statement in your cover letter. Guidelines for resubmitting your figure files are available below the reviewer comments at the end of this letter.

If applicable, we recommend that you deposit your laboratory protocols in protocols.io to enhance the reproducibility of your results. Protocols.io assigns your protocol its own identifier (DOI) so that it can be cited independently in the future. For instructions see: https://journals.plos.org/plosone/s/submission-guidelines#loc-laboratory-protocols. Additionally, PLOS ONE offers an option for publishing peer-reviewed Lab Protocol articles, which describe protocols hosted on protocols.io. Read more information on sharing protocols at https://plos.org/protocols?utm_medium=editorial-email&utm_source=authorletters&utm_campaign=protocols.

As the corresponding author, your ORCID iD is verified in the submission system and will appear in the published article. PLOS supports the use of ORCID, and we encourage all coauthors to register for an ORCID iD and use it as well. Please encourage your coauthors to verify their ORCID iD within the submission system before final acceptance, as unverified ORCID iDs will not appear in the published article. *Only*  the individual author can complete the verification step; PLOS staff *cannot*  verify ORCID iDs on behalf of authors.

We look forward to receiving your revised manuscript.

Kind regards,

JONATHAN BAYUO, PhD

Academic Editor

PLOS One

Journal Requirements:

If the reviewer comments include a recommendation to cite specific previously published works, please review and evaluate these publications to determine whether they are relevant and should be cited. There is no requirement to cite these works unless the editor has indicated otherwise.

Please review your reference list to ensure that it is complete and correct. If you have cited papers that have been retracted, please include the rationale for doing so in the manuscript text, or remove these references and replace them with relevant current references. Any changes to the reference list should be mentioned in the rebuttal letter that accompanies your revised manuscript. If you need to cite a retracted article, indicate the article’s retracted status in the References list and also include a citation and full reference for the retraction notice.

[Note: HTML markup is below. Please do not edit.]

Reviewers' comments:

Reviewer's Responses to Questions

**Comments to the Author**

Reviewer #2: All comments have been addressed

**********

2. Is the manuscript technically sound, and do the data support the conclusions?

Reviewer #2: Yes

**********

3. Has the statistical analysis been performed appropriately and rigorously?

Reviewer #2: Yes

**********

4. Have the authors made all data underlying the findings in their manuscript fully available?

The PLOS Data policy

Reviewer #2: Yes

**********

5. Is the manuscript presented in an intelligible fashion and written in standard English?

Reviewer #2: No

**********

Reviewer #2: Suggestions for improvement

Title it is written as Iranian nurses, consider writing ICU and oncology nurses from Iran

EFA should use oblique rotation instead of Varimax

Perform Parallel Analysis to support the four-factor solution.

Include multigroup CFA or acknowledge this as a limitation.

Explain why criterion validity could not be evaluated because no Persian gold standard exists.

Please justify ML estimation as the items are ordinal

Please maintain consistent capitalization of family-centered care in the manuscript

**********

what does this mean?). If published, this will include your full peer review and any attached files.

If you choose “no”, your identity will remain anonymous but your review may still be made public.

**Do you want your identity to be public for this peer review?**  For information about this choice, including consent withdrawal, please see our Privacy Policy

Reviewer #2: No

**********

[NOTE: If reviewer comments were submitted as an attachment file, they will be attached to this email and accessible via the submission site. Please log into your account, locate the manuscript record, and check for the action link "View Attachments". If this link does not appear, there are no attachment files.]

To ensure your figures meet our technical requirements, please review our figure guidelines: https://journals.plos.org/plosone/s/figures

You may also use PLOS’s free figure tool, NAAS, to help you prepare publication quality figures: https://journals.plos.org/plosone/s/figures#loc-tools-for-figure-preparation.

NAAS will assess whether your figures meet our technical requirements by comparing each figure against our figure specifications.

# Author comment: Author response to Decision Letter 2

**DOI:** 10.1371/journal.pone.0361384.r004

23 Jul 2026

Response to the Editor and Reviewers

We gratefully acknowledge the Editor and reviewers for their valuable comments and constructive feedback. All comments have been carefully addressed, and the corresponding revisions have been incorporated into the revised manuscript. All changes are highlighted using Track Changes. Below, we provide a point-by-point response to each comment.

Sincerely,

Corresponding Author

Author’s responses Comments of Editor

Thank you for this comment. We carefully reviewed all reviewer comments and confirmed that no specific publications were recommended for citation. Nevertheless, we reviewed the recent literature and updated the reference list where appropriate. If the reviewer comments include a recommendation to cite specific previously published works, please review and evaluate these publications to determine whether they are relevant and should be cited. There is no requirement to cite these works unless the editor has indicated otherwise.

Thank you for this valuable comment. We carefully reviewed the entire reference list and confirmed that all references are complete, accurate, and up to date. We also verified that none of the cited articles have been retracted. Minor formatting corrections were made to ensure consistency with the journal style. Please review your reference list to ensure that it is complete and correct. If you have cited papers that have been retracted, please include the rationale for doing so in the manuscript text, or remove these references and replace them with relevant current references. Any changes to the reference list should be mentioned in the rebuttal letter that accompanies your revised manuscript. If you need to cite a retracted article, indicate the article’s retracted status in the References list and also include a citation and full reference for the retraction notice.

Response to Comments of Reviewer 2 Comments of Reviewer 2

1- Thank you for the suggestion. The title has been revised accordingly.

2- Thank you. The EFA was repeated using Direct Oblimin rotation, and the manuscript has been revised accordingly.

3- Thank you. Parallel analysis was performed in R and confirmed the four-factor solution. The results and figure have been added to the manuscript.

4- Thank you. As recommended, the absence of multigroup CFA has been acknowledged in the Limitations section.

5- Thank you. This point has been clarified in the Methods section by stating that no validated Persian gold-standard instrument was available for criterion validity assessment.

6- Thank you. We have added a justification for using maximum likelihood estimation with bootstrapping in the Preliminary Data Checks subsection.

7- Thank you. The terminology has been revised throughout the manuscript to ensure consistent capitalization. Reviewer #2: Suggestions for improvement

1- Title it is written as Iranian nurses, consider writing ICU and oncology nurses from Iran

2- EFA should use oblique rotation instead of Varimax

3- Perform Parallel Analysis to support the four-factor solution.

4- Include multigroup CFA or acknowledge this as a limitation.

5- Explain why criterion validity could not be evaluated because no Persian gold standard exists.

6- Please justify ML estimation as the items are ordinal

7- Please maintain consistent capitalization of family-centered care in the manuscript

Attachment Submitted filename: response_to_reviewers_auresp_2.docx

# Editor report: Decision Letter 2

**DOI:** 10.1371/journal.pone.0361384.r005

**Authors:** Mohammad Saadati

11 Oct 2026

Psychometric Evaluation of the Palliative Nursing Care Quality Scale (PNCQS) Among ICU and Oncology Nurses in Iran

PONE-D-26-23180R2

Dear Dr. Shahmari,

We’re pleased to inform you that your manuscript has been judged scientifically suitable for publication and will be formally accepted for publication once it meets all outstanding technical requirements.

Within one week, you’ll receive an e-mail detailing the required amendments. When these have been addressed, you’ll receive a formal acceptance letter and your manuscript will be scheduled for publication.

An invoice will be generated when your article is formally accepted. Please note, if your institution has a publishing partnership with PLOS and your article meets the relevant criteria, all or part of your publication costs will be covered. Please make sure your user information is up-to-date by logging into Editorial Manager at Editorial Manager® and clicking the ‘Update My Information' link at the top of the page. For questions related to billing, please contact billing support.

If your institution or institutions have a press office, please notify them about your upcoming paper to help maximize its impact. If they’ll be preparing press materials, please inform our press team as soon as possible -- no later than 48 hours after receiving the formal acceptance. Your manuscript will remain under strict press embargo until 2 pm Eastern Time on the date of publication. For more information, please contact onepress@plos.org.

Kind regards,

Mohammad Saadati

Academic Editor

PLOS One

Additional Editor Comments (optional):

Reviewers' comments:

# Editor report: Acceptance letter

**DOI:** 10.1371/journal.pone.0361384.r006

**Authors:** Mohammad Saadati

PONE-D-26-23180R2

PLOS One

Dear Dr. Shahmari,

I'm pleased to inform you that your manuscript has been deemed suitable for publication in PLOS One. Congratulations! Your manuscript is now being handed over to our production team.

At this stage, our production department will prepare your paper for publication. This includes ensuring the following:

* All references, tables, and figures are properly cited

* All relevant supporting information is included in the manuscript submission,

* There are no issues that prevent the paper from being properly typeset

You will receive further instructions from the production team, including instructions on how to review your proof when it is ready. Please keep in mind that we are working through a large volume of accepted articles, so please give us a few days to review your paper and let you know the next and final steps.

Lastly, if your institution or institutions have a press office, please let them know about your upcoming paper now to help maximize its impact. If they'll be preparing press materials, please inform our press team within the next 48 hours. Your manuscript will remain under strict press embargo until 2 pm Eastern Time on the date of publication. For more information, please contact onepress@plos.org.

You will receive an invoice from PLOS for your publication fee after your manuscript has reached the completed accept phase. If you receive an email requesting payment before acceptance or for any other service, this may be a phishing scheme. Learn how to identify phishing emails and protect your accounts at https://explore.plos.org/phishing.

If we can help with anything else, please email us at customercare@plos.org.

Thank you for submitting your work to PLOS One and supporting open access.

Kind regards,

PLOS One Editorial Office Staff

on behalf of

Dr. Mohammad Saadati

Academic Editor

PLOS One
